# Results and lessons learned from the sbv IMPROVER metagenomics diagnostics for inflammatory bowel disease challenge

**DOI:** 10.1038/s41598-023-33050-0

**Published:** 2023-04-18

**Authors:** Lusine Khachatryan, Yang Xiang, Artem Ivanov, Enrico Glaab, Garrett Graham, Ilaria Granata, Maurizio Giordano, Lucia Maddalena, Marina Piccirillo, Ichcha Manipur, Giacomo Baruzzo, Marco Cappellato, Batiste Avot, Adrian Stan, James Battey, Giuseppe Lo Sasso, Stephanie Boue, Nikolai V. Ivanov, Manuel C. Peitsch, Julia Hoeng, Laurent Falquet, Barbara Di Camillo, Mario R. Guarracino, Vladimir Ulyantsev, Nicolas Sierro, Carine Poussin

**Affiliations:** 1 PMI R&D, Philip Morris Products S.A., Quai Jeanrenaud 5, 2000 Neuchâtel, Switzerland; 2 ITMO University, St. Petersburg, Russian Federation; 3 University of Luxembourg, Luxembourg, Luxembourg; 4 Georgetown University, Washington, DC USA; 5 Consiglio Nazionale delle Ricerche, Naples, Italy; 6 University of Padua, Padua, Italy; 7 University of Fribourg, Fribourg, Switzerland

**Keywords:** Clinical microbiology, Metagenomics, Microbiome, Inflammatory bowel disease, Crohn's disease, Ulcerative colitis, Computer science

## Abstract

A growing body of evidence links gut microbiota changes with inflammatory bowel disease (IBD), raising the potential benefit of exploiting metagenomics data for non-invasive IBD diagnostics. The sbv IMPROVER metagenomics diagnosis for inflammatory bowel disease challenge investigated computational metagenomics methods for discriminating IBD and nonIBD subjects. Participants in this challenge were given independent training and test metagenomics data from IBD and nonIBD subjects, which could be wither either raw read data (sub-challenge 1, SC1) or processed Taxonomy- and Function-based profiles (sub-challenge 2, SC2). A total of 81 anonymized submissions were received between September 2019 and March 2020. Most participants’ predictions performed better than random predictions in classifying IBD versus nonIBD, Ulcerative Colitis (UC) versus nonIBD, and Crohn’s Disease (CD) versus nonIBD. However, discrimination between UC and CD remains challenging, with the classification quality similar to the set of random predictions. We analyzed the class prediction accuracy, the metagenomics features by the teams, and computational methods used. These results will be openly shared with the scientific community to help advance IBD research and illustrate the application of a range of computational methodologies for effective metagenomic classification.

## Introduction

Inflammatory bowel disease (IBD) is a group of disorders characterized by chronic inflammation of the gastrointestinal tract. The two main IBD manifestations are ulcerative colitis (UC) and Crohn’s disease (CD). Despite UC and CD differing in their location, histology, and distribution of inflamed areas^1–5^, similarities in symptoms and some disease phenotype overlap make precise, distinct classification difficult^6^. The current diagnostic gold standard is based on histopathologic and endoscopic criteria (lesion pattern and anatomical distribution)^7^. However, differential diagnosis is currently infeasible in up to 10% of IBD patients^5^, making it impossible to plan an appropriate treatment strategy. Thus, while the classical diagnosis – prognosis – treatment paradigm is firmly anchored on the anatomical and pathological classification of CD and UC, identification of new entities or processes involved in IBD pathogenesis, as well as new tools to analyze the resulting data are needed to provide more accurate diagnoses. In this regard, non-invasive, cost-effective, rapid, and reproducible biomarkers would help clinicians diagnose IBD and select appropriate treatment plans for individual patients.

Dysbiosis, defined as an imbalanced gut microbial community, has been consistently reported in IBD patients over the last 15 years^8,9^. Pre-clinical and clinical studies^10^, as well as the recently published data from the Integrative Human Microbiome Project^11^, have revealed distinct IBD metagenomics features, such as a global decrease in biodiversity and lower proportions of Firmicutes and Bacteroidetes relative to those of Proteobacteria and Actinobacteria. Technological advances in DNA sequencing methods, greater data accessibility, and the development of new computational tools for data integration have improved characterization of the gastrointestinal microbiome, highlighting microbiota assessment as a novel tool to support IBD diagnostics and/or prognostics.

Proper and accurate data analysis is crucial to reveal the information contained within a metagenome. The core process for metagenomics analysis is called profiling and is intended to quantify characteristics of metagenomics datasets (hereafter called features). Features can be obtained by applying various reference-based (comparing metagenomics reads to the known sequences) and reference-free analyses, allowing the determination of features using sequencing reads alone. There is a growing number of studies highlighting the potential of both reference-based and reference-free types of features for metagenomics-based IBD diagnostics^12^. However, a systematic investigation comparing different aspects of metagenomics-based diagnostics is still missing.

**Figure 1:**
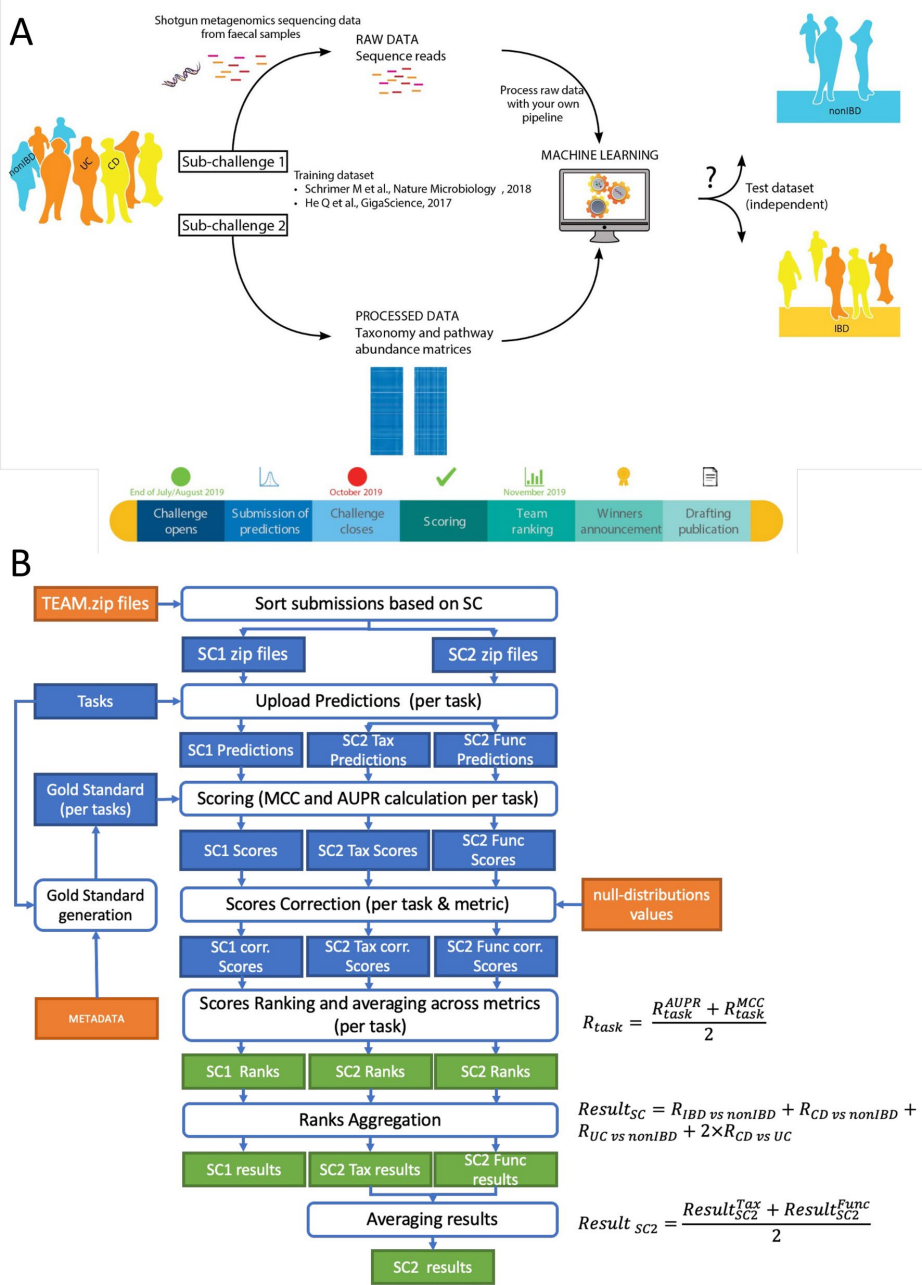
Overview of the metagenomics for IBD diagnosis challenge. (**A**) challenge design, (**B**) challenge scoring schema.

The Metagenomics Diagnosis for Inflammatory Bowel Disease Challenge (herein referred to as the “MEDIC Challenge” or “Challenge”), organized as part of the sbv IMPROVER project^13^, was aimed at investigating the diagnostic potential of metagenomics data in discriminating between IBD patients (UC or CD) and subjects without IBD (nonIBD), and to distinguish between UC and CD subjects among IBD patients (Fig. 1A). The Challenge was organized into two sub-challenges. In the first sub-challenge, “MEDIC RAW” or SC1, participants received shotgun metagenomics sequencing reads from fecal samples of human subjects diagnosed with IBD, including CD and UC, and subjects without IBD. In SC1, participants had the option to process raw metagenomic data with their own analysis pipeline before classifying samples. In the second sub-challenge, “MEDIC PROCESSED” or SC2, participants were provided with taxonomic and functional matrices resulting from the processing of raw metagenomics sequencing reads by the organizers using a standardized pipeline. This enabled participation in the MEDIC Challenge without having to process raw metagenomics data, and therefore without in-depth metagenomics knowledge. Participants could choose to participate in either one or both SCs. The challenge results together with extensive post-challenge analysis allowed for unbiased evaluation of the diagnostics potential of metagenomics data, as well as the assessment of metagenomics profiling techniques and classification pipelines which used various machine learning (ML) approaches.

## Results

### Participation summary and challenge results

Worldwide participation of teams in the MEDIC challenge led to a total of 80 submissions, including 17 and 63 submissions for the SC1 and SC2, respectively. Three submissions for both SC1 and SC2 were deleted as they were fully duplicated. During post-challenge data analysis, a collaboration with an expert research group in the field of metagenomics resulted in three additional submissions for SC1. However, these submissions were not considered as part of the participant submissions and were used to develop a deeper understanding of the scientific problem addressed by the challenge. Thus, the total amount of submissions was equal to 17 (among which 14 were eligible for awarding) in the case of SC1 and 60 in case of SC2 (all eligible for awarding).

**Figure 2:**
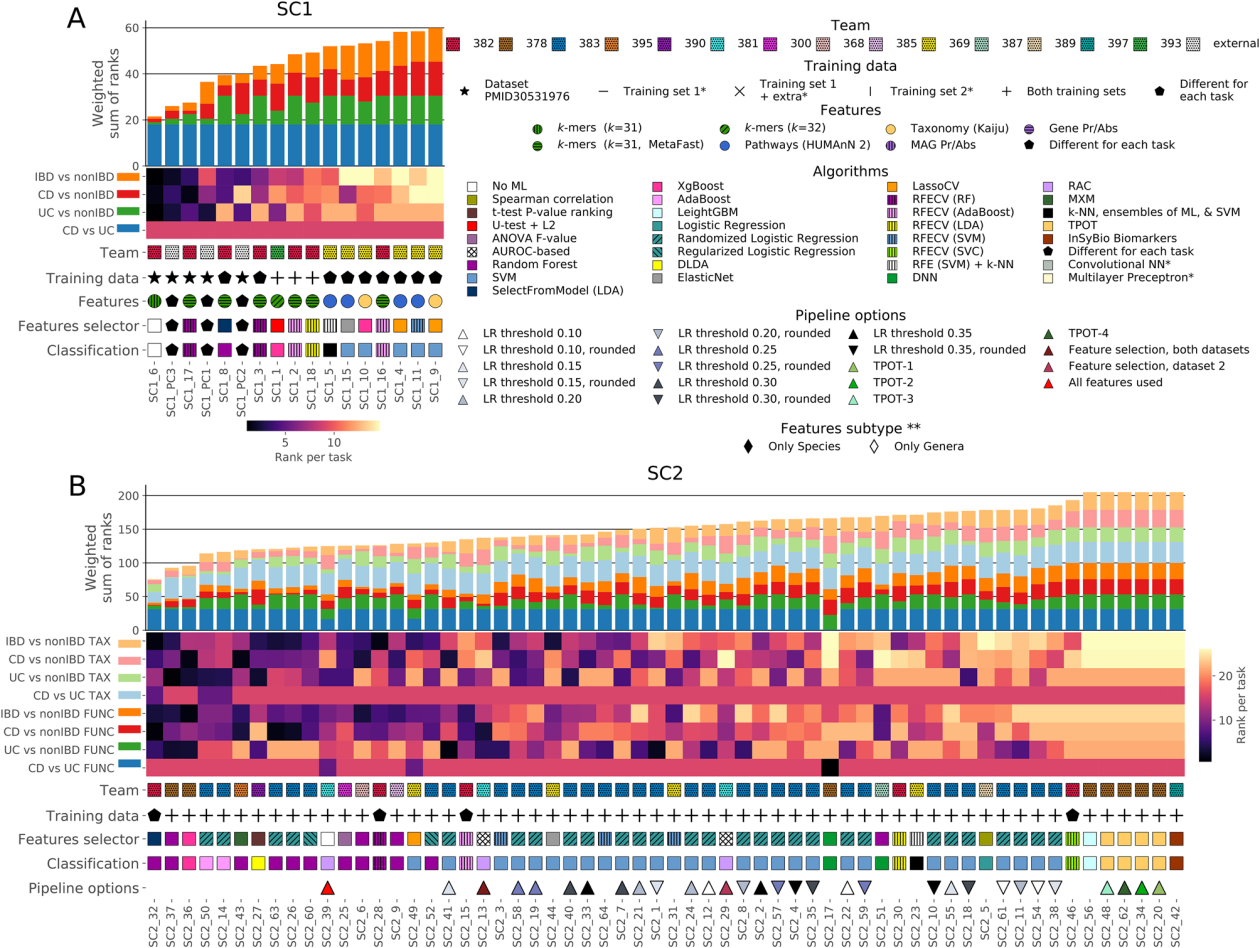
Final submissions ranking in the sbv IMPROVER MEDIC Challenge. Results for SC1(A) and SC2 (B) are shown separately. Bar plot of the weighted sum of ranks (WSR) sorted from the lowest (best) to the highest (worst) WSR. A heatmap shows the WSR stratified by 2-class task and data type (applicable only for SC2 submissions). Submission details such as the submitting team number, feature type (applicable only for SC1 submissions), ML algorithms used for feature selection and classification, and pipeline options are also shown. For optimization purposes, the legend contains additional information not shown on the current summary graphics but required for the interpretation of results in other figures. This and all following figures were generated using Matplotlib python library (version 3.3.3, https://matplotlib.org).

Anonymized participants’ predictions submitted for each SC were independently scored according to the strategy defined before challenge closure (Fig. 1B and detailed description in “Methods” section). Briefly, two complementary metrics—Matthews’ correlation coefficient (MCC) and the the area under the precision recall (AUPR)—were computed by comparing participants’ predictions in the form of confidence values [0,1], reflecting the probability that a sample belongs to group 1, with the gold standard (true class labels of the test dataset). This comparison was made for all four pairwise classification tasks and data types (Taxonomy and Function for SC2 only)*.* Participants’ predictions were considered non-significant when MCC and AUPR scores were lower than the 95th percentile of MCC and AUPR values from distributions obtained with 10 000 random predictions (Supplementary Fig. 1; here and after called the null distribution). MCC and AUPR values were then converted into ranks and aggregated as a weighted sum of ranks (WSR) as described in Fig. 1B. Overall, the ranking of final participants’ submissions for SC1 and SC2 is shown in Fig. 2, and detailed scores are provided in Supplementary Table 1. After the review and acceptance of the scoring results by an independent, external expert panel, the identities of the top three winning teams for each SC were disclosed^14^.

### Post-challenge analysis

#### Metagenomics data are informative to discriminate IBD versus nonIBD subjects but insufficient for UC versus CD distinction

The majority of submitted predictions, regardless of the SC, successfully classified “IBD versus nonIBD” better than random (Table 1, Supplementary Fig. 1), providing significant (higher than the 95^th^ percentile of the corresponding null distribution) MCC and/or AUPR values.

**Table 1:** MEDIC prediction statistics.

Sub-challenge	Task	Total submissions	Performance better than random		
			MCC	AUPR	MCC and AUPR
SC1	IBD versus nonIBD	17	12	11	10
	CD versus nonIBD		13	10	8
	UC versus nonIBD		8	6	6
	CD versus UC		0	0	0
SC2 Taxonomy	IBD versus nonIBD	60	52	36	36
	CD versus nonIBD		50	36	35
	UC versus nonIBD		29	22	18
	CD versus UC		3	0	0
SC2 Function	IBD versus nonIBD	60	40	31	26
	CD versus nonIBD		38	21	16
	UC versus nonIBD		29	23	17
	CD versus UC		3	1	1

The number of submissions with MCC and/or AUPR significantly better than random was larger for the “CD versus nonIBD” classification task compared with the “UC versus nonIBD” task. This may be due to a training set imbalance, as there were more CD than UC samples in the training dataset. The discrimination of CD and UC samples within the IBD group was a more challenging task (Table 1). Indeed, none of the SC1 submissions performed significantly better than random. In the case of SC2, three submissions based on Taxonomy and Function data types (not matching among data types) had significant MCCs. Among those, only one submission (based on Function data type) also showed a significant AUPR. Thus, the number of submissions with MCC or AUPR better than 95th percentile of the corresponding null distribution for “CD versus UC” task (3 out of 60) was not higher than in a setting where all submissions were randomly generated.

#### Tree-based ML approaches along with reference-free features demonstrated the best overall performance

The scoring results (Fig. 2, more details per-task visualization in Supplementary Fig. 2), combined with the characteristics of the participants’ computational approaches used to tackle the challenge, enabled a visualization of key observations made in the scope of the post-challenge analysis regarding effective classification strategies.

SC1 participants had the freedom to process raw metagenomics data with their own analysis pipeline, as well as the possibility to use additional training datasets. Features produced by SC1 raw metagenomics data analysis pipelines were either reference-based (Taxonomic or Functional profiles, with feature generation algorithms different from ones used by the challenge organizers for SC2 data generation) or reference-free (k-mers of various length, Metagenome-Assembled Genomes (MAGs) or Metagenome-Assembled Genes). The use of reference-free features and an external dataset for model training was associated with higher classification performance in comparison with standard reference-based features in the case of SC1 (Fig. 2A, Supplementary Fig. 2A-D).

The best-performing submission for SC1 (SC1 submission 6, Fig. 2A) involved a simple statistical technique for sample labelling. The classification approach was based on the search of unique “discriminative” k-mers in the training data. The subsequent sample class label decision was based on the comparison of the proportions of k-mers from each group found in the sample. Except for this latter submission done in the context of SC1, all other top-performing predictions for both SC1 and SC2 challenges used tree-based ML methods (e.g., random forest (RF) and various boosting approaches) for sample classification. This conclusion on the superiority of tree-based methods was based on the final aggregation score and was consistent across different tasks of SC1 (Supplementary Fig. 2A-D). However, for SC2, the performance of algorithms varied depending on the task and data type (Supplementary Fig. 2E-L).

#### IBD samples were more often misclassified than nonIBD samples

Box plots of confidence values (Supplementary Fig. 3) showed clear separation between the IBD and nonIBD groups of samples for the submissions with highest performance (with significant MCC and/or AUPR values) and no clear separation for submissions with the lowest performance. The class separation was especially pronounced for the “IBD versus nonIBD” task (Supplementary Fig. 3A, E, and I) and was not observed in the “CD versus UC” task (Supplementary Fig. 3D, H, and L). The same figure shows that most predictors classified samples more consistently as nonIBD, thus increasing the false positive rate for IBD classification. However, there are several exceptions (e.g., SC1 submission 17 and SC2 Taxonomy submission 37). Finally, several SC2 submissions (mostly belonging to one team) demonstrated predictions better than random across different tasks in the case of inverted sample labels.

**Figure 3:**
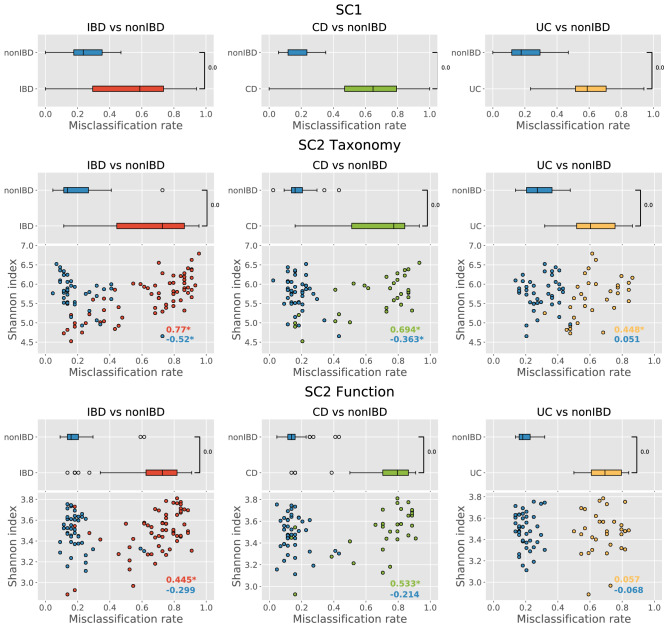
Distribution of sample misclassification rates stratified by group. Results for different SC, data types (for SC2), and 2-class tasks are shown separately. Results for SC2 also have additional panels representing the correlation between samples’ misclassification rate and diversity. Samples are stratified by group. Correlation coefficients between misclassification rate and diversity are shown on the lower right corner of each SC2-related plots. *next to the correlation coefficient implies *P* value < 0.05.

Sample misclassification was investigated more closely for each SC, data type (in the case of SC2), and task using the binarization of confidence values to allocate a sample to one or the other class. To avoid biases, only 44 out of 60 SC2 submissions were considered for this task since 16 pairs of SC2 submissions (all provided by the same team) were identical regarding binarized predictions (but different regarding confidence values) due to the significant similarities in their classification algorithms and the fact that the same features were used for the model training (Supplementary Figs. 4–6, Supplementary Table 1). As shown in Fig. 3, the level of misclassification rate for IBD samples was statistically higher compared with that of nonIBD samples. The same figure shows the correlation between the misclassification rate and the Shannon diversity index of samples. In the case of SC2, the correlation coefficient was positive for IBD samples and negative for nonIBD samples.

#### The best predictive models for discriminating IBD versus nonIDB subjects were characterized by specific combinations between Taxonomy or Function features and ML algorithms

One of the key findings from the previous sbv IMPROVER Systems Toxicology challenge was that feature selection is a key step to build a performant predictive model. Indeed, once relevant discriminative features have been selected, the impact of ML methods’ performance did not reveal significant differences on the final performance^15^.

To verify whether this observation was also true for the MEDIC challenge, seven popular ML methods (hereafter called the in-house ML pipeline)—k-nearest neighbor (kNN), linear discriminant analysis (LDA), RF, support vector machine with linear kernel (SVMlinear), partial least squares discriminant analysis (PLS-DA), naïve Bayes (NB), and extreme gradient boosting (XGBoost)—were used to assess the performance of Taxonomy- and Function-based discriminative signatures selected by SC2 participants for the “IBD versus nonIBD” task. As described earlier, only 44 out of 60 SC2 submissions were analyzed given that 16 pairs of submissions had identical signatures.

**Figure 4:**
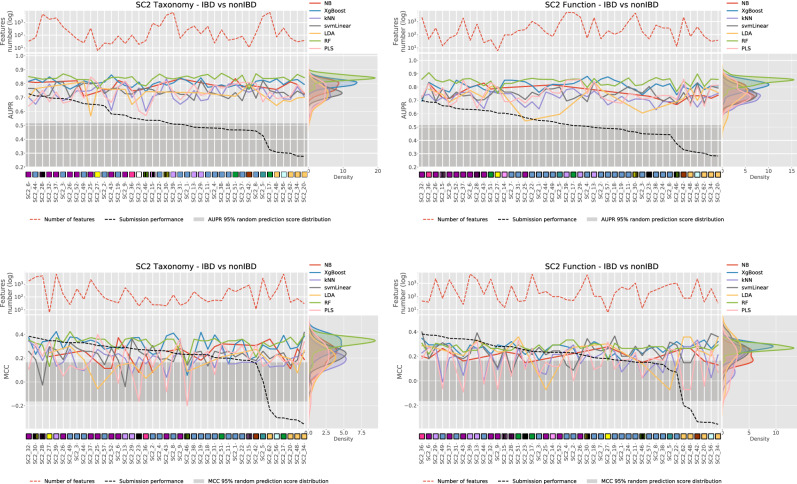
Robustness of individual SC2 “IBD versus nonIBD” signatures controlled using seven in-house ML methods. Results for each data type and metric are shown separately. Submissions are sorted from the best to the worst based on the challenge performance for the relevant data type. Individual performances are shown with dashed black line, performances of each of the seven in-house ML methods are shown with colored lines. The classification algorithm used in each individual submission is shown using the square symbol, the legend can be found on the Fig. 2. The top panel for each graphics represents the number of features per signature in a log10 scale. Finally, the right panel represents the distribution of scores obtained when using individual signatures per ML method.

As shown in Fig. 4, no significant correlation was found between participants’ model performance and the performance obtained using in-house ML pipeline in combination with participants’ Taxonomy- or Function-based signatures. There was also no significant correlation between the number of features selected and the performance (either demonstrated by participants’ ML algorithms or the in-house ML pipeline). Thus, the success of the best-performing SC2 “IBD versus nonIBD” submissions was attributed to the specific combination between the selected features and the ML algorithm used to solve the task. Interestingly, among in-house ML methods, tree-based methods (RF and XGBoost) demonstrated higher predictive performance (both metrics) than the other tested ML methods using participants’ Taxonomy- and Function-based signatures.

#### AUPR obtained with consensus Taxonomy and Function signature-based predictive models outperformed AUPR resulting from individual participants’ signature-based predictions

**Figure 5:**
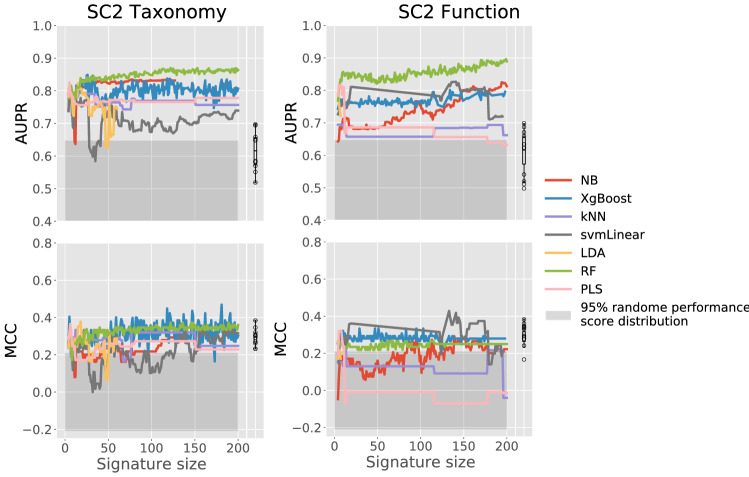
Classification performance of SC2 consensus signatures tested using seven in-house ML methods. Results are shown per metric and per SC2 data type. Each graphic also includes the boxplot showing the metric distribution for the individual SC2 predictions obtained for “IBD versus nonIBD” task and each data type.

**Figure 6:**
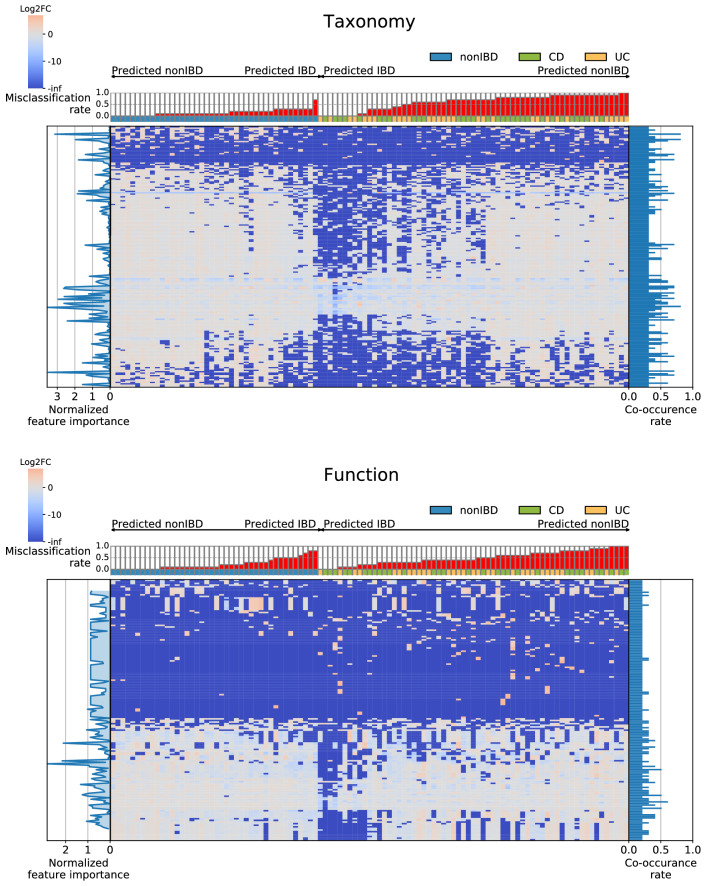
Log2-fold change abundance of the consensus signature features in the test samples. Results for Taxonomy and Function data types are shown separately. Samples are sorted first per label (nonIBD or IBD) and then based on the misclassification rate within each sample group.

The consensus signatures were built by combining the most commonly occurring Taxonomy- or Function-based features across the top-10 best SC2 submissions (only for “IBD versus nonIBD” task). There were 197 signatures generated with numbers of features ranging from 4 to 200. In general, increasing the size of consensus signatures increased the performance (AUPR) of predictive models built using seven in-house ML methods (Fig. 5). The AUPR performance increase was particularly pronounced when using an RF classification algorithm. Overall, the performance of tree-based methods (RF and XGBoost) was higher than that of other ML approaches when using consensus signatures. The abundance of the features included in consensus signatures in the test samples was visualized using the Log2-fold-change relative to the average abundance of the feature across all test samples. A high heterogeneity rate in consensus signature abundance connected to sample misclassification can be observed in Fig. 6.

#### Comparison of the SC2 taxonomic consensus signature and k-mer-based signature showed low overlaps

The discriminative features (k-mers, k = 31) identified by the best performer (SC1 submission 6) for the SC1 “IBD versus nonIBD” task were translated into a k-mer-based Taxonomic signature (see Methods). Shortly, reads containing discriminative k-mers for each group (CD, UC, and nonIBD) were extracted and went through the taxonomic annotation using the same pipeline as for SC2 Taxonomy data creation with the final annotation at species level (Table 2). The final set of obtained species was split into three groups: species associated exclusively with IBD (included CD and/or UC reads), nonIBD, and those commonly shared between the IBD and nonIBD groups. The k-mer-based taxonomic signature was compared to the largest (200 taxa, 120 of which were on species level) SC2 consensus taxonomic signature.

**Table 2:** Taxonomic annotation of k-mer-based reads. Only k-mers identified by the best SC1 performer were used for reads selection.

Group	Number of k*-*mers	Reads extracted	Reads classified
CD	2 926 215 368	9 762 179	3 369 427 (34.52%)
UC	3 044 646 402	80 967	9 526 (11.77%)
nonIBD	2 559 326 025	3 436 809	9 829 (0.29%)

**Figure 7:**
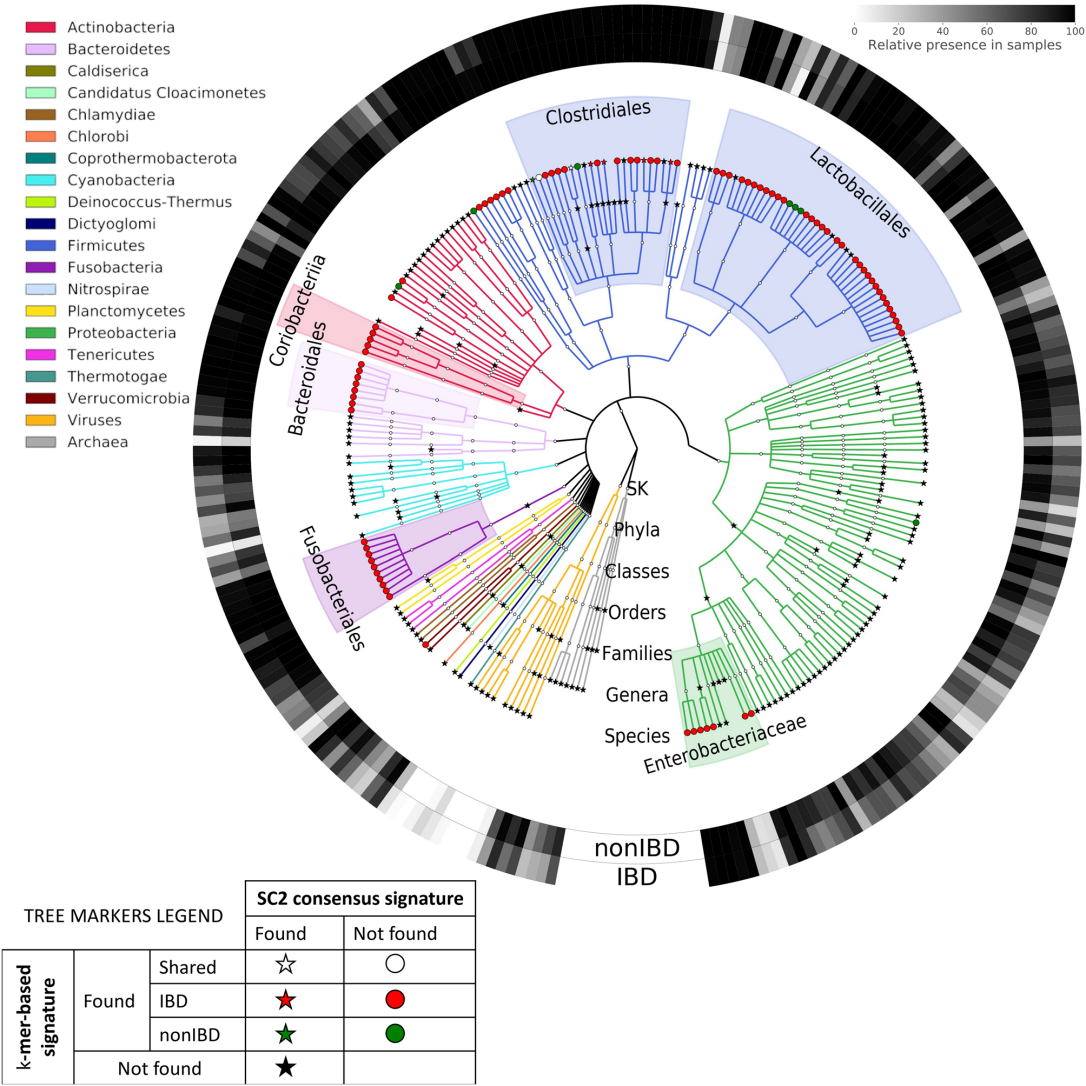
Taxonomic tree showing SC2 Taxonomy consensus signature- and SC1 k-mer-based taxa. Branches are colored per super-kingdom and per phyla for the Bacteria super-kingdom. The taxon origin is represented by the node symbol. For each tree leaf, the proportion of nonIBD and IBD test samples in which the taxon was detected is shown in the first and second outer rings, respectively.

The union of both signatures is visualized as taxonomic tree in Fig. 7, which shows little overlap between signatures at the species and genera levels. Similarly to what was previously observed, we identified seven major phyla represented by the taxa from either signature that were detected in the vast majority of samples: Firmicutes, Proteobacteria, Bacteroidetes, Actinobacteria, Fusobacteria, Cyanobacteria, and Verrucomicrobia. The classical IBD dysbiotic feature at the phylum level has been observed with increased abundance of Proteobacteria, Actinobacteria, and Fusobacteria and decreased abundance of Firmicutes, Bacteroidetes, and Verrucomicrobia^10,16^. Taxa from 12 other bacterial phyla, viruses, and archaea were usually only detected in a small proportion of samples and were predominantly covered by the SC2 consensus signature.

There are many molecular mechanisms connected to IBD-related microbiome alteration. For this reasons, the study analysis paid specific attention to bacteria involved in the most studied of these processes (short chain fatty acid (SCFA) production; pro-or anti-inflammatory cytokine mediation; butyrate, formate, acetate and folate production; host immune response mediation; hydrogen metabolism, etc.). Table 3 summarizes the information about bacterial taxa detected in both signatures with a previously described connection to IBD.

**Table 3:** Taxa detected in SC2 taxonomic consensus signature and k-mer-based signature connected to IBD through the literature review. Taxonomy levels are marked as: Class (C), Order (O), Family (F), Genus (G), Species (S), Strain (STR).

Relevant tax group	Signature	Connection to IBD
FIRMICUTES, Clostridia order		
G: Roseburia, Ruminococcus, Eubacterium, Faecalibacterium	k-mer & SC2	Short Chain Fatty Acids (SFCA) production^17^
S: *R.* *hominis*, *A.* *hardus*	k-mer & SC2	Butyrate production^18–20^
F: Eubacteriaceae		
S: *F. prausnitzii*	k-mer &SC2	Butyrate and anti-inflammatory cytokine production, suppression of pro-inflammatory cytokines^18^
S: *C. difficile*	k-mer	IBD-induced (due to bile acids inhibition) growth causing IBD symptoms and complications^21,22^
FIRMICUTES, Lactobacillales order		
Order in general	k-mer	IBD-associated relative abundance change^23^
S: *L. gazeri*	k-mer	Improves colitis symptoms in mice^24^
S: *L. rhamnosus*, *E. faecium, L. plantarum*, *L. acidophilus*	k-mer	Multi-strain probiotic associated with decreased inflammation in patients with UC, but not in CD^25^
PROTEOBACTERIA		
G: Klebsiella, Salmonella S: *E. coli*	k-mer & SC2	Pro-inflammatory and colitogenic pathobionts^26–29^
G: Pseudomonas	SC2	Pro-inflammation (epithelial cell damage) agent^30^
G: Campylobacter	SC2	Pro-inflammatory cytokines production^31^
STR: cytogenic strains of Class Alphaproteobacteria	SC2	Antagonizing Lachnospiraceae family, thus increasing IBD symptoms^32^
C: Bettaproteobacteria	k-mer	IBD-associated relative abundance change^33^
BACTEROIDETES		
O: Flavobacteriales, Cytophagales	SC2	Decreased abundance is associated with IBD status. Through the sphingolipids production influences the severity of intestinal inflammation and alters host ceramide pools^34–37^
O: Bacteroidales	k-mer	
S: *B. fragilis*, *B. vulgatus*	k-mer	Attenuates pathogenic bacteria-induced colitis^38,39^
STR: *B. fragilis (*enterotoxigenic strains)	k-mer	Increases inflammation by producing certain toxins and pro-inflammatory cytokines^40^
S: *B.* *longum*	k-mer	Immune responses induction and regulation; inflammatory cytokines expression reduction^41^
S: *B.* *adolescentis*	k-mer	Folate productions (reduces the inflammation)^42^
Coriobacteriaceae		Lactate, formate, acetate, and hydrogen sulfate metabolism regulation^43–45^
Eggerthellaceae		IBD-associated relative abundance change^46^
FUSOBACTERIUM		
S: *F.* *nucleatum*	k-mer	Promotion of proinflammatory cytokine secretion and thus damaging the intestinal barrier^47^
CYANOBACTERIA	SC2	IBD-associated relative abundance change^48^
VERRUCOMICROBIA		
G: Akkermansia Verrucomicrobium	SC2	SCFA-producing, decreased relative abundance in IBD subjects^49,50^
S: *A. muciniphilia*	SC2	Colonic mucus restoration^50^

Interestingly, there are notable differences between signatures (Fig. 7): most species included in the SC2 consensus signature already differentiate the IBD and nonIBD groups simply by their presence or absence. This observation is specifically pronounced for the phyla Actinobacteria, Cyanobacteria, Bacteroidetes, and Proteobacteria. Finally, genera differentiating both IBD groups of samples from nonIBD samples obtained during an independent analysis of the challenge test set (published shortly after the challenge closure^51^) have large overlap with the k-mer-based taxonomic signature.

### Wisdom of the crowd

The “Wisdom of the crowd” phenomenon refers to the theory that the collective knowledge of a community (or the aggregation of solutions) is greater than the knowledge of any individual (or individual solution)^52^. We investigated whether consensus classification based on several submitted predictions was more accurate than any of the individual classification submissions. However, prior to this analysis, the list of submissions was alternated to avoid over-weighting particular feature-selecting and classification algorithms. For this purpose, one random submission was selected for each group of submissions performed by the same team and using same feature-selection and classification algorithms. Additionally, submissions identified as inverted—where across several 2-class tasks the inverted-labeled predictions have demonstrated classification quality better than random—were also excluded from the analysis. This resulted in 17 submissions for SC1 and 25 submissions for SC2 Taxonomy and SC2 Function. The complete list of selected submissions for consensus classification can be found in Supplementary Table 2.

The aggregation strategy leading to the incorporation of several individual predictions into one is described in the Methods section and schematically represented on Supplementary Fig. 7. Briefly, the confidence values of the aggregated predictions were calculated per sample as the average of the confidence values for that sample in all incorporated individual predictions.

#### On average, randomly aggregated predictions performed better than individual predictions even when integrating small sets of individual predictions

**Figure 8:**
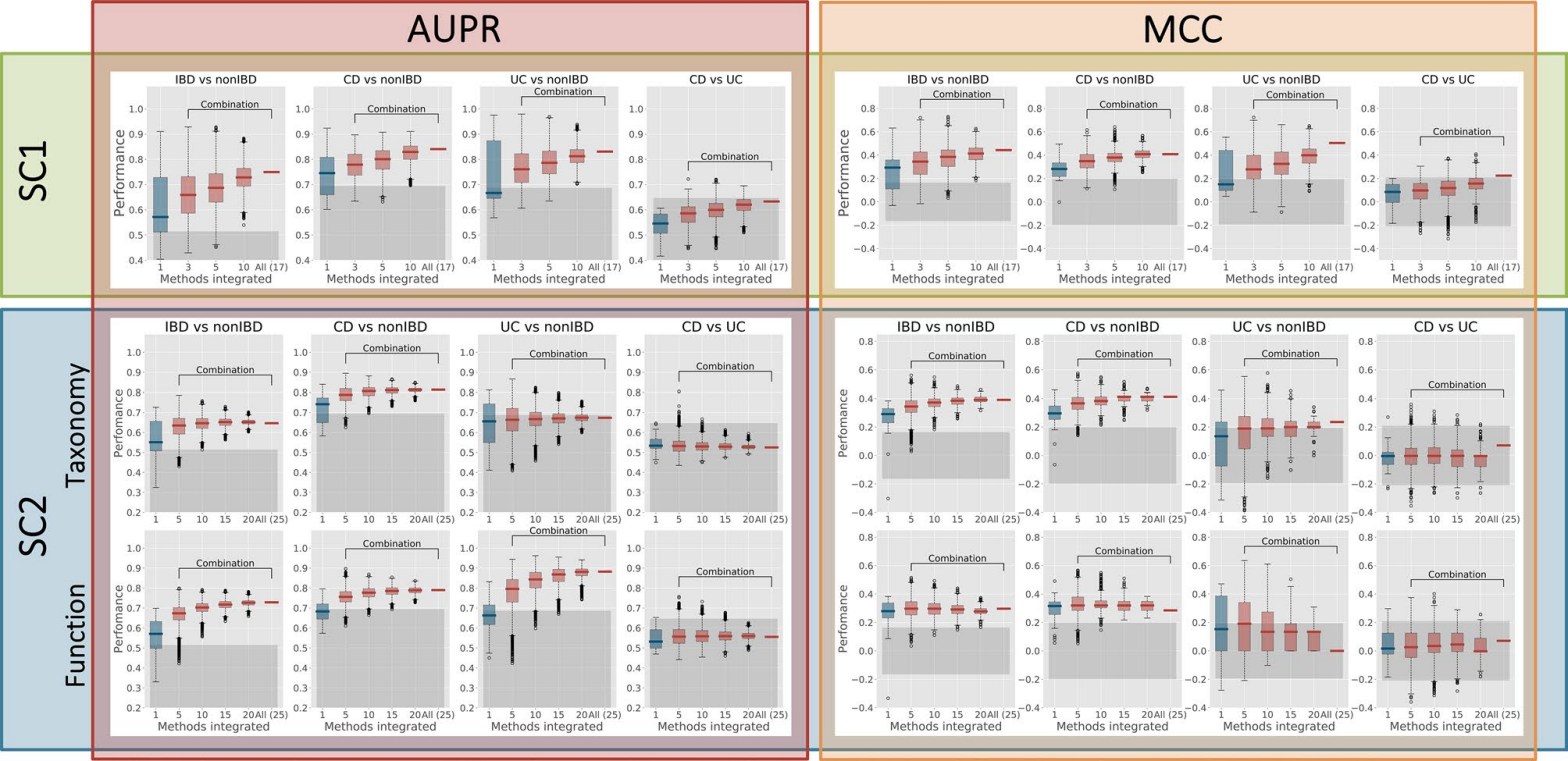
Comparison of the performance of assembly-based versus individual predictions. Results for different SCs, data types, 2-class tasks, and metrics are shown separately. The first boxplot depicts the performance distribution of individual methods. Further boxplots represent the performance when integrating > 1 randomly sampled methods. The last boxplot demonstrates one value obtained after integrating all the individual methods.

A set of aggregated predictions incorporating randomly selected 3, 5, 10, and all 17 submissions for SC1 (5, 10, 20, 15, and all 25 submissions for SC2) was created. In cases where the number of all possible random combinations exceeded 1 000, only 1 000 randomly selected combinations were used. The classification performances of aggregated and individual predictions were compared. As shown in Fig. 8, on average, aggregation-based methods performed better than individual approaches, even when integrating small sets of individual predictions (e.g., just three). Performance increased further with a larger number of integrated methods (except for SC2 Function, MCC, “UC versus nonIBD” task). However, it is important to note that the aggregation-based prediction including all the individual submissions rarely out-performed the best of the individual ones.

Reference-based analysis of the metagenomics data usually implies two different but complementary directions for the dataset description: the understanding of “who is there” (Taxonomic profiling) and “what are they doing” (Functional profiling). Each of the SC2 submissions included the individual Taxonomy- and Function-based predictions for all four binary classification tasks. This provided an opportunity to compare them in a pairwise manner and assess the classification performance of the aggregated prediction. In the first analysis, the results indicated that aggregating Taxonomy- and Function-based prediction is useful. Paired-sample Wilcoxon signed-rank test showed that for three 2-class binary classification tasks, aggregating Taxonomy- and Function-based predictions provided a statistically superior or similar performance than each of the integrated predictions separately (Supplementary Fig. 8). However, more detailed analysis demonstrated that this effect only persists when the difference in individual performances between Taxonomy- and Function-based predictions is small. Once one of the predictions is significantly superior to the other (especially if the better performing one is Function-based), the aggregation of predictions will have a lower performance in comparison with the strongest performer (Supplementary Fig. 9).

#### Aggregation-based predictions are robust to random noise

Robustness of the aggregated prediction to the inclusion of a subset of poorly performing individual methods was investigated using the following approach. All but the three best and three worst predictions were integrated to create a so-called “initial” assembly. One-by-one, the worst three or best three methods were added to form additional combinations, whose performances were investigated using MCC and AUPR metrics. In case of AUPR, adding poor predictions (which usually introduce noise) did not affect the classification quality of the “initial” assembly of methods. On the other hand, additional integration of the best predictions increased the performance of the aggregation-based approach, although not always reaching the predictive performance of the best of the individual methods (Supplementary Fig. 10).

## Discussion

Recent progress in metagenomics and next-generation sequencing methods created the prospect of using the human microbiome as a widespread diagnostics tool. One major challenge is the data complexity coupled with the broad range of computational approaches for data analysis that necessitates developing accurate and independent methods and results verification. Crowdsourcing was shown to be a powerful tool for solving many computational and biological problems^53–57^, specifically for independent verification of methods, results, and conclusions. Here, we discuss the results from the crowdsourced sbv IMPROVER MEDIC computational challenge designed to investigate the diagnostics potential of metagenomics data in the scope of IBD. The challenge was opened to the scientific community between September 2019 and March 2020. There were several studies dedicated to microbiome-based IBD diagnostics prior to the MEDIC challenge; however, most of these works are at risk of bias since the same sample cohort is used for algorithm training and testing, introducing potential limitations with generalizability^12^. In the scope of the MEDIC challenge, participants were offered the option to train their prediction models on two different IBD cohorts and apply it on the third one, thus removing the cohort bias. Participants of the challenge could apply their own metagenomics processing pipeline to obtain microbiome-based features and then develop predictive models or use already computed standard metagenome-associated features to apply their predictive model on them. This allowed us to compare the quality of diagnosis based on different types of microbiome-based features. Finally, the challenge scoring strategy used two complementary metrics, allowing better result interpretation.

Metagenomics data were sufficiently informative to classify IBD and nonIBD samples, but were not sufficient for differentiating between CD and UC using metagenomics data only. From one side, this can be explained by insufficiency of metagenomics data to solve challenging task of CD and UC classification. In that case, incorporation of other omics- and meta-omics data types as well as clinical predictors (age, sex, body mass index, etc.) might be necessary to build discriminative models. Alternatively, since many recent studies suggested additional heterogeneity within patients diagnosed with CD or UC, the differentiation problem might be caused by an inaccurate gold standard. In that case, the IBD categorization may require further refinement analysis of larger patient datasets aimed to identify potential disease sub-types that could thus improve the quality of “CD versus UC” predictions.

The use of reference-free metagenomics features was associated with better classification outcomes compared with standard reference-based features. Reference-free features incorporate all the genomic information in analyzed datasets since they do not rely on often incomplete reference databases. However, utilizing reference-free features produces sparse data that is difficult to analyze and biologically interpret. Thus, the introduction of the reference-free yet biologically interpretable features for metagenomics data profiling (e.g., using catalogs of MAGs or species, k-mer agglomerations, etc.) could be a promising direction for the further development of non-invasive IBD diagnostics.

Overall, tree-based ML classification algorithms demonstrated better performance compared with other types of classification techniques. This observation is consistent with previous metagenomic-based IBD diagnostics studies^58–61^. Tree-based ML approaches involve hyper-rectangular partitioning of the feature space and may therefore reflect complex patterns specific to smaller sub-regions of the feature space more adequately than linear ML approaches. We noticed, however this was not always true for certain data types and tasks. Particularly, the “UC versus nonIBD” task is best tackled by using Function-based data in combination with different linear regression ML algorithms.

None of the signatures developed by the participants in the scope of SC2 “IBD versus nonIBD” task could demonstrate robust performance when applying a set of different classification algorithms, demonstrating that prediction success relies on the combination between the classification algorithm and the set of features. In contrast to the original hypothesis that the set of features is primary to the classification algorithm^15^, we observed that across all participants’ signatures in the scope of SC2 for the “IBD versus nonIBD” task, tree-based algorithms (RF and XGBoost) were associated with higher classification accuracy. This supports the previous conclusion about the superior performance of tree-based algorithms for the task of metagenomic-based IBD diagnostics.

Using participants’ signatures, we designed consensus signatures containing the most co-occurring features among the top-10 Taxonomy and Function-based submissions. Application of the consensus signatures did improve prediction quality in comparison with individual participants’ signatures. We observed little overlap between the SC2 consensus and SC1 k-mer-based taxonomic signatures. This can be explained by the different training data and approaches used to generate Taxonomic features. Despite the low overlap, the union of signatures was enriched with taxa previously reported to be associated with IBD.

The results revealed that IBD samples were misclassified more often than nonIBD samples. Further investigation identified a connection of samples’ misclassification and diversity: IBD samples with high diversity and nonIBD samples with low diversity had high misclassification rates. This relationship was especially pronounced for the taxa included in the SC2 taxonomic consensus signature. At the same time, taxa from the SC2 consensus signature had high abundance variability in IBD samples compared with nonIBD samples, and this observation could explain the higher misclassification rate of IBD samples. The higher the diversity of IBD samples, the more frequently IBD samples tend to be misclassified. Indeed, these latter IBD samples tend to show taxa abundance profiles similar to those of nonIBD samples. It is important to note that recent studies observed variability of diversity across different IBD samples^62,63^ and even stressed the necessity of considering samples’ diversity when performing IBD sample classification^11^. We believe that the influence of sample diversity, as well as possible pre-classification diversity-based samples grouping, might be beneficial for metagenome-based IBD diagnostics.

Our research revealed special advantages in aggregating individual predictions to improve the classification quality. This “Wisdom of the crowd” effect was previously described for various system biology studies^64–66^. We experimented with aggregating different predictions within one data type, as well as aggregating results of the same predictive model applied to different data types. On average, aggregating predictions within the same data type randomly demonstrated better classification than individual predictions, although the aggregation-based prediction did not always outperform the best of the individual ones. Additionally, the aggregation of predictions was robust to the inclusion of the random noise. This observation suggests that in the case of no prior knowledge of the algorithm’s performance, it can be beneficial to use different algorithms for the subsequent prediction aggregation. A similar conclusion was reached for the aggregation of predictions obtained using same algorithm but different reference-based data types.

Several new non-invasive routines for IBD diagnostics have been proposed in recent years. Fecal samples collection followed by the analysis of stool IBD biomarkers—especially fecal calprotectine (FCP)—demonstrates both sensitivity and specificity to IBD (https://doi.org/10.3389/fmed.2022.920732, https://doi.org/10.3109/00365521.2014.987809). Despite the presence of well-established routines like FCP testing, the development of microbiome-based diagnostics is highly important for better disease understanding and subsequent possible treatment. Also, the diagnostic potential of the human microbiome will continually increase with the number of sequenced IBD samples. Finally, merging different non-invasive diagnostic approaches by performing a multi-omics analysis and thus combining signals originating from the different marker types could potentially improve non-invasive diagnostics in the future. It is also important to note that despite being the most informative non-invasive technique, fecal sampling has high patient non-compliance, which hampers the translatability of fecal IBD biomarkers research to clinical practice^67–70^. This problem should be addressed by better pre-sampling education of the patients.

Overall, we believe that coupling the power and wisdom of the crowd with the independent and unbiased evaluation of computational methods increased knowledge in the field of microbiome-based IBD diagnostics and revealed potential directions for its further development.

## Conclusions

- The diagnostic potential of metagenomics data was shown to be sufficient to classify IBD and nonIBD samples, but not CD and UC samples.
- Overall, the use of reference-free metagenome profiling and tree-based classification algorithms were associated with better classification outcomes.
- The combination of discriminative features determined by the most successful predictions was enriched with organisms for with strong connections with IBD have been previously reported.
- IBD samples were statistically more frequently misclassified than nonIBD samples, with sample misclassification strongly connected to their taxonomical and functional alpha diversity.
- The assessment of individual and aggregated predictions demonstrated that in case of no prior knowledge of the algorithms’ classification performance, it can be beneficial to aggregate the predictions from multiple different algorithms.

## Methods

### Specific tasks

Participants of both SCs were asked to tackle four binary classification problems by classifying samples as follows:

- IBD (class 1) versus nonIBD (class 2)
- UC (class 1) versus nonIBD (class 2)
- CD (class 1) versus nonIBD (class 2)
- UC (class 1) versus CD (class 2)

Participants developed classification models for the four binary classification problems using training datasets recommended by the challenge organizers and/or their private dataset (only for SC1) and then applied their models to the test dataset. For each sample of the test dataset, participants were asked to provide a confidence value, ranging between 0 (lowest confidence) and 1 (highest confidence), reflecting the estimated probability that a sample belongs to the class 1 of the certain problem.

SC2 participants were additionally asked to provide the list of selected features (a subset of TaxIDs or PathIDs) used in their classification prediction model(s) applied on the test dataset, and their associated value of importance (optional).

Finally, challenge participants were asked to describe their classification approaches by providing information to allow reproducibility.

### Challenge data

For both SCs, the organizers proposed that the participants use shotgun metagenomics sequencing data from two previously published human studies^71,72^ as training datasets, and the challenge organizers provided a test dataset from an unpublished (at the time of competition) human study^51^. The datasets were provided as quality controlled raw and processed data as described below. The class labels associated with test dataset samples constituted the gold standard against which participants’ submissions were scored.

#### Training datasets

The overview of the training datasets given to participants is shown in Table 4. Challenge organizers provided participants with class labels associated with selected samples from each of the training datasets. The metadata extracted from the original publications associated with the training datasets can be found in the Supplementary Table 3. Participants were free to use additional publicly available data.

**Table 4:** MEDIC Challenge training datasets.

Dataset	Original publication	Country of origin	Total samples	Samples selected	Inclusion criteria	nonIBD	CD	UC
1	^14^	USA	1338	54	QC, one sample per person	14	23	17
2	^15^	China	123	116	QC	53	63	0

#### Test dataset

The sbv IMPROVER test dataset consisted of 105 paired-end whole genome sequencing of faecal samples from patients with CD or UC and nonIBD individuals living in the republic of Tatarstan (Russian Federation).

The testing dataset was not publicly available during the Challenge. A detailed description of the study design and generation of the metagenomics sequencing dataset were previously published^51^. The study was reviewed and approved by the local ethics committee of the Kazan Federal University, Kazan, Russia. All methods were carried out in accordance with the ethical standards laid down in the 1964 Declaration of Helsinki and its later amendments. Written informed consent was obtained from all study participants before enrollment.

#### Sample quality checking and selection

The quality control (QC) pipeline described below was used to assess the quality of samples from each dataset. The reasons behind using this pipeline were to filter out potential human and technical contaminating sequences and confirm the good overall quality of the remaining sequencing reads.

Raw reads were mapped to the human genome (hg38) using Minimap2 (version 2.8) aligner^73^ with options –a (Concise Idiosyncratic Gapped Alignment Report and output alignment in SAM format) and –x sr (short single-end reads without splicing). Unmapped reads were collected using the SAMtools (version 1.7) *view*^74^ command with the –f 12 SAM flag (both paired reads are unmapped). Obtained reads were subjected to contaminants and adapter trimming using the BBDUKprogram of the BBTools toolkit (version 37.99)^75^ with the *k* size set to 23. QC reports for the raw, pre-, and post-trimming reads were generated using FastQC (version 0.11.6) software^76^ and collated using the MultiQC (version 1.7) module for Python^77^. Sample selection was based on the samples’ metadata and the results of the QC pipeline (Table 5).

**Table 5:** Criteria used for sample selection.

Dataset	Provided for	Reads pairs in the post-QC data	Subjects’ age	Additional criteria
Training dataset 1	Training	> 10 × 10^6^	≥ 18 years old	One time-point sample (the earliest one) per subject
Training dataset 2	Training	> 20 × 10^6^	≥ 18 years old	Included samples that are not marked as “host contaminated” in the original research metadata
sbv IMPROVER	Testing	> 20 × 10^6^	≥ 18 years old	Confirmed IBD diagnosis as CD or UC

#### Generation of Taxonomy and functional abundance matrices

Samples that passed the QC were used to generate Taxonomy and Functional abundance matrices for all three datasets (both training datasets and the testing dataset).

Taxonomic classification of reads was performed for each sample using Kraken2^78^ and abundance re-estimation at the Species, Genus, Family, Order, Class, Phylum, and Superkingdom levels using Bracken (version 2.0^79^). The Kraken2 reference database was built using the *-standard* option that enforces the download of the RefSeq^80^ bacteria/archaeal genomes, RefSeq plasmid sequences, RefSeq complete viral genomes, and GRCh38 human genome (database built in February 2019). The Bracken database was built using a read length of 100 bp for the training datasets, 75 bp for the testing dataset, and k-mer length of 35 bp. Bracken correction was performed with the minimum of 10 reads required for classification at the specified taxonomic rank. For each dataset, sample-associated relative abundance profiles were organized as a taxonomy matrix. Each taxonomy matrix was distributed in a tab-separated file. The column names represented the sample identification number. The first column contained the TaxID associated with relative abundances reported for each sample at Species, Genus, Family, Order, Class, Phylum, and Superkingdom levels. The relative abundances (ranging from 0 to 100%) calculated for a sample corresponded to the percentage of reads assigned to a specific taxon, relative to the total number of reads classified for all taxons at a specific taxonomy level. In addition to the taxonomy abundance matrices, Challenge participants were provided with a “TaxID description” file that contained the taxonomy rank and full name associated with each TaxID.

Functional matrices for SC2 were generated using pathways abundances. Starting from the raw reads, pathway abundances matrices were generated using the Biobakery’s “wmgx” pipeline^81^ using default settings and reference databases. An exception was the 16S database that was generated using a text search for “16S” in the NCBI nucleotide database; selecting all sequences belonging to “Fungi”, “Protists”, “Bacteria”, “Archaea”, and “Viruses”; with a range of length between 700 and 2000 bp; and storing those sequences into a FASTA file. More specifically, the HUMAnN2 component of the Biobakery pipeline computed pathway abundances for each sample by associating reads with MetaCyc reaction pathways, stratified where possible by species. Pathway abundance files generated for each sample using the Biobakery pipeline were joined into a single matrix with the sample identification numbers as column names and the unique pathway identification number as row names. When pathway abundance was missing for a sample, the pathway abundance value was set to 0.

In addition to the pathway abundances matrices, Challenge participants were provided with a “PathID description” file that contained the full pathway information associated with each PathID.

### Scoring procedure

The schematic representation of the scoring procedure is shown in Fig. 1B. The scoring procedure is described below in detail.

#### Scoring participant submissions

For each SC and 2-class binary classification problem, anonymized participant predictions were scored against the gold standard corresponding to the true class labels of samples from the testing dataset using the MCC and the AUPR curve metrics. These metrics are complementary since MCC is threshold dependent while the AUPR is threshold independent. Samples for which the label was not part of the gold standard for the 2-class binary classification problem under evaluation were ignored for both metrics’ calculations.

For each sample, participants provided confidence values P_x_ (x = 1 or 2 for class 1 and class 2, respectively) that a sample belongs to class 1 (P_1_) or class 2 (P_2_ = 1 − P_1_). The class confidence values P_x_ ranged between 0 and 1, with 1 being the most confident and 0 the least confident.

For MCC calculation, a confusion matrix was built using a fixed threshold of 0.5 to binarize the predictions between class 1 and class 2. For AUPR calculation, confusion matrices were built using a moving threshold from 1 to 0.5. At each threshold value, recall and specificity were calculated and used to build the AUPR curve and calculate the area under the curve as a score of prediction performance.

Each SC included four 2-class binary classification problems: (1) IBD versus nonIBD, (2) CD versus nonIBD, (3) UC versus nonIBD, and (4) CD versus UC. For SC2, the predictions for all four 2-class binary classification problems were based on the provided input taxonomy and function abundance matrices. Thus, participants had to generate four and eight predictions for SC1 and SC2, respectively. Based on participant submissions, the MCC and AUPR scores were computed.

#### Score significance evaluation

**Figure 9:**
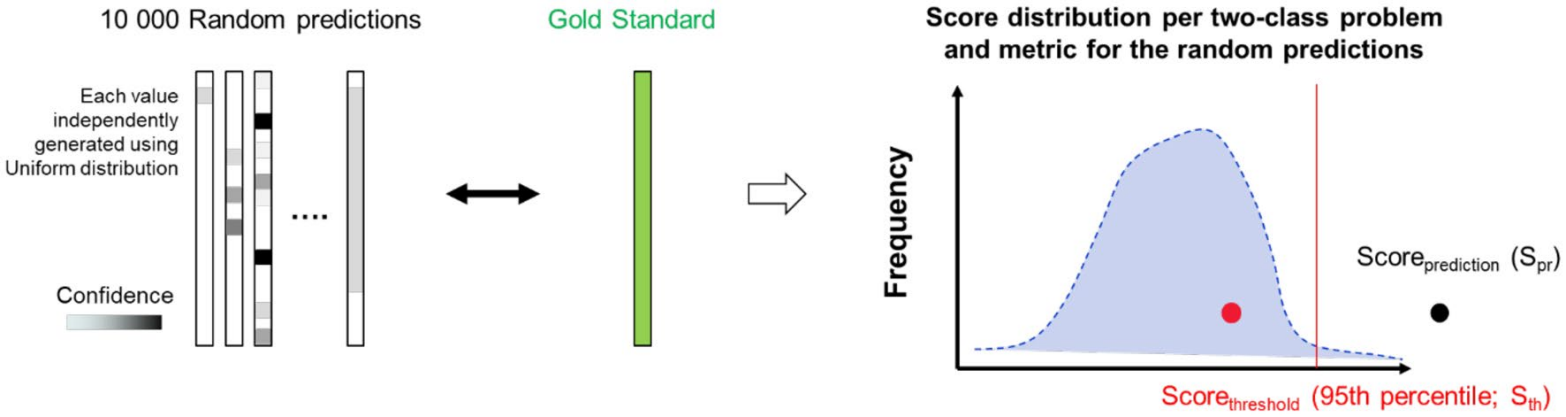
Overview of prediction randomness evaluation.

To assess if a prediction was better than random, distributions of scores from random predictions were generated. For that, a confidence value between 0 and 1 (from the uniform distribution) was assigned independently to each of 105 samples (test set size). Ten thousand sets of 105 samples were generated as random predictions. For each random prediction and 2-class problem, the MCC and AUPR were computed, considering only samples for which the label was part of the gold standard for the 2-class binary classification problem under evaluation. The score distributions of 10 000 random predictions were generated for each of the 2-class problems and metrics. Participant scores were compared with the random score distribution. Scores greater than the value at the 95^th^ percentile (threshold) of the random prediction score distribution were considered as significant. Scores smaller than the value at the 95^th^ percentile of the random prediction score distribution were considered to not be better than random, and their value was set to the score obtained at the 95^th^ percentile of the random prediction score distribution (Fig. 9).

### Score aggregation and final submission and team ranking

To determine the teams with the overall best performance for each SC, scores were aggregated as follows:

- For each metric and 2-class problem, scores were ranked across teams (the highest score gets the lowest rank) using the “rank” function of *pandas.DataFrame* package for Python with the option method set to *average* (i.e., it assigns the average of ranks to the similar values).
- For each 2-class problem and submission, ranks across different metrics were averaged:
  $${R}_{problem}={\frac{{R}_{problem}^{AUPR}+{R}_{problem}^{MCC}}{2}}$$
- For each submission, the aggregation of results consisted in a weighted sum of ranks (WSR) giving more weight to the “CD versus UC” 2-class problem that was more challenging.

For SC1:

$$WSR_{{SC1}} = R_{{IBD\;vs\;nonIBD}} + R_{{CD\;vs\;nonIBD}} + R_{{UC\;vs\;nonIBD}} + 2 \times R_{{CD\;vs\;UC}}$$

For SC2, the final WSR was calculated as an average across the values obtained for two abundance matrices.

$$\begin{aligned} WSR_{{SC2}} & = \frac{1}{2} \times \left\{ {\left( {R_{{IBD\;vs\;nonIBD}} + R_{{CD\;vs\;nonIBD}} + R_{{UC\;vs\;nonIBD}} + 2 \times R_{{CD\;vs\;UC}} } \right)_{T} } \right. \\ & \quad + \left. {\left( {R_{{IBD\;vs\;nonIBD}} + R_{{CD\;vs\;nonIBD}} + R_{{UC\;vs\;nonIBD}} + 2 \times R_{{CD\;vs\;UC}} } \right)_{F} } \right\} \\ \end{aligned}$$

For each SC, the top three teams with the lowest WSR were declared as the best-performing teams after final review and approval by the Scoring Review Panel.

### Sample misclassification

Misclassification analysis identified samples that were misclassified in each participants’ submission. Rates of misclassification for each sample were calculated as the proportion of submissions for which this sample was incorrectly classified relative to the total number of non-replicated submissions (some teams provided multiple submissions with exactly the same predictions after binarizing confidence values, although the confidence values themselves were different. In this case, only one submission was kept and used to calculate the sample misclassification rate). This calculation was performed independently for each SC and 2-class problem.

### Statistical and mathematical tools

Within-sample diversity was estimated with the Shannon index (H′) using the following formula:

$${H}^{^{\prime}}=- \sum_{i=1}^{R}{p}_{i}*\mathrm{ln}{p}_{i}$$

where *p*_*i*_ represents the relative abundance of the i-th taxa or i-th pathway.

Statistical analysis for the independent set of values was performed using non-parametric Mann–Whitney U tests. Statistical hypothesis testing for dependent sets of values were tested using Wilcoxon signed-rank tests.

### SC2 consensus signature generation

A consensus signature analysis was performed only for the "IBD versus nonIBD" task of SC2. The 10 best submissions based on the rank across different metrics were selected separately for Taxonomy- and Function-based predictions without considering predictions with duplicated absolute values. Within each prediction, features were ranked according to the importance value provided by the participants, and ranks across all selected predictions were averaged to obtain final rank for each feature. Top-200 features were selected for both Taxonomy and Function data types as a SC2 consensus signatures.

### SC1 k-mer-based Taxonomic signature generation

The discriminative k-mers (k = 31) identified by the best SC1 performer (SC1 submission 6) for the SC1 “IBD versus nonIBD” task were processed to obtain biologically interpretable features. Discriminative k-mers were split into three groups: unique for CD, UC, and nonIBD groups of samples in the training data. Reads containing discriminative k-mers for each group (CD, UC, and nonIBD) were extracted and went through taxonomic annotation using Kraken2^74^ with the following abundance re-estimation at the Species level using Bracken (version 2.0^75^). Results for the CD and UC groups were further merged into results of the IBD group.

### In-house ML pipeline

Individual SC2 “IBD versus nonIBD” signatures as well as SC2 consensus signatures were evaluated using in-house ML pipeline including seven different ML classifiers: NB, XGBoost, kNN, svmLinear, LDA, RF, PLS-DA. For the performance evaluation MCC and AUPR were used. Seven different ML were chosen based on the methods submitted by different teams and literature^15^. The R package caret with version 6.0.81^82^ provides a universe interface to use the above methods. The default parameters in caret were used. We directly used the XGBoost package rather than the wrapper function xgbTree in package caret because there is a parallel issue for xgbTree in caret. Five-fold cross-validation with 10 times repeats were used to obtain the performance in cross-validation.

### Aggregation-based predictions creation

Aggregated predictions were created by averaging the confidence value per sample across individual predictions selected to be aggregated (Supplementary Fig. 7).

### Ethics approval and consent to participate

Any individual or team had to register in the sbv IMPROVER platform to participate and access the challenge data. By registering, participants agreed to comply with the terms and conditions of the challenge. Data submitted by challenge participants were anonymized during their upload on the website. The scoring was conducted blindly following a strict procedure detailed in the “Scoring methodology” section of the Methods.

## Supplementary Information

Supplementary Information 1.

Supplementary Information 2.

Supplementary Information 3.

Supplementary Information 4.

Supplementary Information 5.

## Data Availability

Sequencing reads for the test dataset were deposited on the Sequence Read Archive and available under project number PRJNA893901 (https://www.ncbi.nlm.nih.gov/bioproject/PRJNA893901). Challenge submissions (per-task and data type confidence values) can be found in Supplementary Table 4.

## Abbreviations

AUPR: Area under the precision recall
CD: Crohn’s disease
IBD: Inflammatory bowel disease
kNN: K-nearest neighbor
LDA: Linear discriminant analysis
MCC: Matthews’ correlation coefficient
MEDIC: Metagenomics diagnosis for inflammatory bowel disease challenge
ML: Machine learning
NB: Naive Bayes
PLS-DA: Partial least squares discriminant analysis (PLS-DA)
RF: Random forest
SC1: Sub-challenge 1
SC2: Sub-challenge 2
svmLinear: Support vector machine with linear kernel
UC: Ulcerative colitis
WSR: Weighted sum of ranks
XGBoost: EXtreme gradiant boosting

## References

[1] Baumgart DC, Sandborn WJ (2007). Inflammatory bowel disease: Clinical aspects and established and evolving therapies. Lancet.

[2] Baumgart DC (2009). The diagnosis and treatment of Crohn’s disease and ulcerative colitis. Deutsches Aerzteblatt Online.

[3] Conrad K, Roggenbuck D, Laass MW (2014). Diagnosis and classification of ulcerative colitis. Autoimmun. Rev..

[4] Laass MW, Roggenbuck D, Conrad K (2014). Diagnosis and classification of Crohn’s disease. Autoimmun. Rev..

[5] Tontini GE (2015). Differential diagnosis in inflammatory bowel disease colitis: State of the art and future perspectives. World J. Gastroenterol..

[6] Bernstein CN, Fried M, Krabshuis JH, Cohen H, Eliakim R, Fedail S (2010). World gastroenterology organization practice guidelines for the diagnosis and management of IBD in 2010. Inflamm. Bowel Dis..

[7] Annese V, Daperno M, Rutter MD, Amiot A, Bossuyt P, East J (2013). European evidence based consensus for endoscopy in inflammatory bowel disease. J. Crohn’s Colitis.

[8] Ott SJ (2004). Reduction in diversity of the colonic mucosa associated bacterial microflora in patients with active inflammatory bowel disease. Gut.

[9] Manichanh C (2006). Reduced diversity of faecal microbiota in Crohn’s disease revealed by a metagenomic approach. Gut.

[10] Frank DN, St. Amand AL, Feldman RA, Boedeker EC, Harpaz N, Pace NR (2007). Molecular-phylogenetic characterization of microbial community imbalances in human inflammatory bowel diseases. Proc. Natl. Acad. Sci..

[11] Lloyd-Price J, Arze C, Ananthakrishnan AN, Schirmer M, Avila-Pacheco J, Poon TW (2019). Multi-omics of the gut microbial ecosystem in inflammatory bowel diseases. Nature.

[12] Gubatan J, Levitte S, Patel A, Balabanis T, Wei MT, Sinha SR (2021). Artificial intelligence applications in inflammatory bowel disease: Emerging technologies and future directions. World J. Gastroenterol..

[13] Meyer P, Hoeng J, Rice JJ, Norel R, Sprengel J, Stolle K (2012). Industrial methodology for process verification in research (IMPROVER): Toward systems biology verification. Bioinformatics.

[14] MEDIC. https://www.intervals.science/resources/sbv-improver/medic.

[15] Belcastroa V, Poussin C, Xiang Y, Giordano M, Tripathi KP, Boda A (2018). The sbv IMPROVER Systems Toxicology computational challenge: Identification of human and species-independent blood response markers as predictors of smoking exposure and cessation status. Comput. Toxicol..

[16] Vich Vila A, Imhann F, Collij V, Jankipersadsing SA, Gurry T, Mujagic Z (2018). Gut microbiota composition and functional changes in inflammatory bowel disease and irritable bowel syndrome. Science Translational Medicine..

[17] Parada Venegas D (2019). Short chain fatty acids (SCFAs)-mediated gut epithelial and immune regulation and its relevance for inflammatory bowel diseases. Front. Immunol..

[18] Machiels K, Joossens M, Sabino J, De Preter V, Arijs I, Eeckhaut V (2013). A decrease of the butyrate-producing species *Roseburia hominis* and Faecalibacterium prausnitziidefines dysbiosis in patients with ulcerative colitis. Gut.

[19] Facchin S (2020). Microbiota changes induced by microencapsulated sodium butyrate in patients with inflammatory bowel disease. Neurogastroenterol. Motil..

[20] Kang S, Denman SE, Morrison M, Yu Z, Dore J, Leclerc M (2010). Dysbiosis of fecal microbiota in Crohnʼs disease patients as revealed by a custom phylogenetic microarray. Inflamm. Bowel Dis..

[21] Zhang L, Liu F, Xue J, Lee SA, Liu L, Riordan SM (2022). Bacterial species associated with human inflammatory bowel disease and their pathogenic mechanisms. Front. Microbiol..

[22] Sorg JA, Sonenshein AL (2008). Bile salts and glycine as cogerminants for clostridium difficile spores. J. Bacteriol..

[23] Xu X (2022). The gut metagenomics and metabolomics signature in patients with inflammatory bowel disease. Gut Pathogens.

[24] Han DH (2022). Co-administration of Lactobacillus gasseri KBL697 and tumor necrosis factor-alpha inhibitor infliximab improves colitis in mice. Sci. Rep..

[25] Bjarnason I, Sission G, Hayee B (2019). A randomised, double-blind, placebo-controlled trial of a multi-strain probiotic in patients with asymptomatic ulcerative colitis and Crohn’s disease. Inflammopharmacology.

[26] Baldelli V, Scaldaferri F, Putignani L, Del Chierico F (2021). The role of enterobacteriaceae in gut microbiota dysbiosis in inflammatory bowel diseases. Microorganisms.

[27] Garrett WS, Gallini CA, Yatsunenko T, Michaud M, DuBois A, Delaney ML (2010). Enterobacteriaceae Act in concert with the gut microbiota to induce spontaneous and maternally transmitted colitis. Cell Host Microbe.

[28] Ruby T, McLaughlin L, Gopinath S, Monack D (2012). Salmonella’s long-term relationship with its host. FEMS Microbiol. Rev..

[29] Geddes K, Rubino S, Streutker C, Cho JH, Magalhaes JG, Le Bourhis L (2010). Nod1 and Nod2 regulation of inflammation in the salmonella colitis model. Infect. Immun..

[30] Deng Q, Barbieri JT (2008). Molecular mechanisms of the cytotoxicity of ADP-ribosylating toxins. Annu. Rev. Microbiol..

[31] Mahendran V, Riordan SM, Grimm MC, Tran TAT, Major J, Kaakoush NO (2011). Prevalence of campylobacter species in adult Crohn’s disease and the preferential colonization sites of campylobacter species in the human intestine. Heimesaat MM, editor. PLoS ONE.

[32] Sun D, Bai R, Zhou W, Yao Z, Liu Y, Tang S (2020). Angiogenin maintains gut microbe homeostasis by balancing α-Proteobacteria and Lachnospiraceae. Gut.

[33] Jangid A, Fukuda S, Seki M, Horiuchi T, Suzuki Y, Taylor TD (2020). Association of colitis with gut-microbiota dysbiosis in clathrin adapter AP-1B knockout mice. Blachier F, editor. PLoS ONE.

[34] Stojanov S, Berlec A, Štrukelj B (2020). The influence of probiotics on the firmicutes/bacteroidetes ratio in the treatment of obesity and inflammatory bowel disease. Microorganisms.

[35] Alam MT (2020). Microbial imbalance in inflammatory bowel disease patients at different taxonomic levels. Gut Pathogens.

[36] Eckburg PB (2005). Diversity of the human intestinal microbial flora. Science.

[37] Brown EM, Ke X, Hitchcock D, Jeanfavre S, Avila-Pacheco J, Nakata T (2019). Bacteroides-derived sphingolipids are critical for maintaining intestinal homeostasis and symbiosis. Cell Host Microbe.

[38] Waidmann M, Bechtold O, Frick J, Lehr H, Schubert S, Dobrindt U (2003). Bacteroides vulgatus protects against Escherichia coli-induced colitis in gnotobiotic interleukin-2-deficient mice. Gastroenterology.

[39] Round JL, Mazmanian SK (2009). The gut microbiota shapes intestinal immune responses during health and disease. Nat. Rev. Immunol..

[40] Rabizadeh S, Rhee K-J, Wu S, Huso D, Gan CM, Golub JE (2007). Enterotoxigenic *Bacteroides fragilis*: A potential instigator of colitis. Inflamm. Bowel Dis..

[41] Yao S, Zhao Z, Wang W, Liu X (2021). Bifidobacterium longum: Protection against inflammatory bowel disease. Wang K, editor. J. Immunol. Res..

[42] Pompei A, Cordisco L, Amaretti A, Zanoni S, Matteuzzi D, Rossi M (2006). Folate production by bifidobacteria as a potential probiotic property. Appl. Environ. Microbiol..

[43] Zhao X, Zhang Z, Hu B, Huang W, Yuan C, Zou L (2018). Response of gut microbiota to metabolite changes induced by endurance exercise. Front. Microbiol..

[44] Clavel T, Desmarchelier C, Haller D, Gérard P, Rohn S, Lepage P (2014). Intestinal microbiota in metabolic diseases. Gut Microbes..

[45] Mottawea P (2016). Altered intestinal microbiota–host mitochondria crosstalk in new onset Crohn’s disease. Nat. Commun..

[46] Edwards J-A, Tan N, Toussaint N, Ou P, Mueller C, Stanek A (2020). Role of regenerating islet-derived proteins in inflammatory bowel disease. World J. Gastroenterol..

[47] Dharmani P, Strauss J, Ambrose C, Allen-Vercoe E, Chadee K (2011). Fusobacterium nucleatum infection of colonic cells stimulates MUC2 mucin and tumor necrosis factor alpha. Bäumler AJ, editor. Infect. Immun..

[48] Santoru ML (2017). Cross sectional evaluation of the gut-microbiome metabolome axis in an Italian cohort of IBD patients. Sci. Rep..

[49] Chen T, Wang R, Duan Z, Yuan X, Ding Y, Feng Z (2021). Akkermansia muciniphila protects against psychological disorder-induced gut microbiota-mediated colonic mucosal barrier damage and aggravation of colitis. Front. Cell. Infect. Microbiol..

[50] Qian K, Chen S, Wang J, Sheng K, Wang Y, Zhang M (2022). A β-N-acetylhexosaminidase Amuc_2109 from Akkermansia muciniphila protects against dextran sulfate sodium-induced colitis in mice by enhancing intestinal barrier and modulating gut microbiota. Food Funct..

[51] Lo Sasso G, Khachatryan L, Kondylis A, Battey JND, Sierro N, Danilova NA (2020). Inflammatory bowel disease-associated changes in the gut: Focus on Kazan patients. Inflamm. Bowel Dis..

[52] Yi SKM, Steyvers M, Lee MD, Dry MJ (2012). The wisdom of the crowd in combinatorial problems. Cogn. Sci..

[53] Good BM, Su AI (2013). Crowdsourcing for bioinformatics. Bioinformatics.

[54] Talikka M, Bukharov N, Hayes WS, Hofmann-Apitius M, Alexopoulos L, Peitsch MC (2017). Novel approaches to develop community-built biological network models for potential drug discovery. Expert Opin. Drug Discov..

[55] Sparks R, Lau WW, Tsang JS (2016). Expanding the immunology toolbox: Embracing public-data reuse and crowdsourcing. Immunity.

[56] Shah N, Levy AE, Moriates C, Arora VM (2015). Wisdom of the crowd. Acad. Med..

[57] Linde J, Schulze S, Henke SG, Guthke R (2015). Data- and knowledge-based modeling of gene regulatory networks: An update. EXCLI J..

[58] Bakir-Gungor B, Hacılar H, Jabeer A, Nalbantoglu OU, Aran O, Yousef M (2022). Inflammatory bowel disease biomarkers of human gut microbiota selected via different feature selection methods. PeerJ.

[59] LaPierre N, Ju CJ-T, Zhou G, Wang W (2019). MetaPheno: A critical evaluation of deep learning and machine learning in metagenome-based disease prediction. Methods.

[60] Pasolli E, Truong DT, Malik F, Waldron L, Segata N (2016). Machine learning meta-analysis of large metagenomic datasets: Tools and biological insights. PLoS Comput. Biol..

[61] Eck A, de Groot EFJ, de Meij TGJ, Welling M, Savelkoul PHM, Budding AE (2017). Robust microbiota-based diagnostics for inflammatory bowel disease. McAdam AJ, editor. J. Clin. Microbiol..

[62] Mirsepasi-Lauridsen HC, Vranckx K, Nielsen HV, Andersen LO, Archampong T, Krogfelt KA (2022). Substantial intestinal microbiota differences between patients with ulcerative colitis from Ghana and Denmark. Front. Cell. Infect. Microbiol..

[63] Mirsepasi-Lauridsen HC, Vrankx K, Engberg J, Friis-Møller A, Brynskov J, Nordgaard-Lassen I (2018). Disease-specific enteric microbiome dysbiosis in inflammatory bowel disease. Front. Med..

[64] Marbach D, Costello JC, Küffner R, Vega NM, Prill RJ, Camacho DM (2012). Wisdom of crowds for robust gene network inference. Nat. Methods.

[65] Stolovitzky G, Prill RJ, Califano A (2009). Lessons from the DREAM2 challenges. Ann. N. Y. Acad. Sci..

[66] Papin JA, Mac GF (2019). Wisdom of crowds in computational biology. PLoS Comput. Biol..

[67] Buisson A, Gonzalez F, Poullenot F, Nancey S, Sollellis E, Fumery M (2017). Comparative Acceptability and Perceived Clinical Utility of Monitoring Tools. Inflamm. Bowel Dis..

[68] Kalla R, Boyapati R, Vatn S, Hijos G, Crooks B, Moore GT (2018). Patients’ perceptions of faecal calprotectin testing in inflammatory bowel disease: Results from a prospective multicentre patient-based survey*. Scand. J. Gastroenterol..

[69] Maréchal C, Aimone-Gastin I, Baumann C, Dirrenberger B, Guéant J, Peyrin-Biroulet L (2017). Compliance with the faecal calprotectin test in patients with inflammatory bowel disease. United Eur. Gastroenterol. J..

[70] Khakoo NS (2021). Patient adherence to fecal calprotectin testing is low compared to other commonly ordered tests in patients with inflammatory bowel disease. Crohn’s Colitis 360.

[71] He Q (2017). Two distinct metacommunities characterize the gut microbiota in Crohn’s disease patients. GigaScience.

[72] Schirmer M, Franzosa EA, Lloyd-Price J, McIver LJ, Schwager R, Poon TW (2018). Dynamics of metatranscription in the inflammatory bowel disease gut microbiome. Nat. Microbiol..

[73] Li H (2018). Minimap2: Pairwise alignment for nucleotide sequences. Birol I, editor. Bioinformatics.

[74] Li H, Handsaker B, Wysoker A, Fennell T, Ruan J, Homer N (2009). The sequence alignment/map format and SAMtools. Bioinformatics.

[75] BBMap. SourceForge. http://sourceforge.net/projects/bbmap.

[76] Andrews, S. Babraham bioinformatics—FastQC A quality control tool for high throughput sequence data (2010). http://www.bioinformatics.babraham.ac.uk/projects/fastqc.

[77] Ewels P, Magnusson M, Lundin S, Käller M (2016). MultiQC: Summarize analysis results for multiple tools and samples in a single report. Bioinformatics.

[78] Wood DE, Lu J, Langmead B (2019). Improved metagenomic analysis with Kraken 2. Genome Biol.

[79] Lu J, Breitwieser FP, Thielen P, Salzberg SL (2017). Bracken: Estimating species abundance in metagenomics data. PeerJ Comput. Sci..

[80] O’Leary NA, Wright MW, Brister JR, Ciufo S, Haddad D, McVeigh R (2015). Reference sequence (RefSeq) database at NCBI: Current status, taxonomic expansion, and functional annotation. Nucleic Acids Res..

[81] McIver LJ, Abu-Ali G, Franzosa EA, Schwager R, Morgan XC, Waldron L (2017). bioBakery: A meta’omic analysis environment. Hancock J, editor. Bioinformatics.

[82] Kuhn, M., *et al*. caret: Classification and Regression Training. R-Packages. 2020. https://cran.r-project.org/web/packages/caret/index.html.

