# Supplementary material for: Results and lessons learned from the sbv IMPROVER metagenomics diagnostics for inflammatory bowel disease challenge
Source: Sci Rep. 2023 Apr 18;13:6303. doi: 10.1038/s41598-023-33050-0 (PMC10113391; doi:10.1038/s41598-023-33050-0)

Supplementary Figure 1. Classification scores for the submitted predictions relatively to the distribution of scores obtained for 10 000 random predictions. Results are shown separately for each SC, data type (for SC2), 2-class problem, and metric.

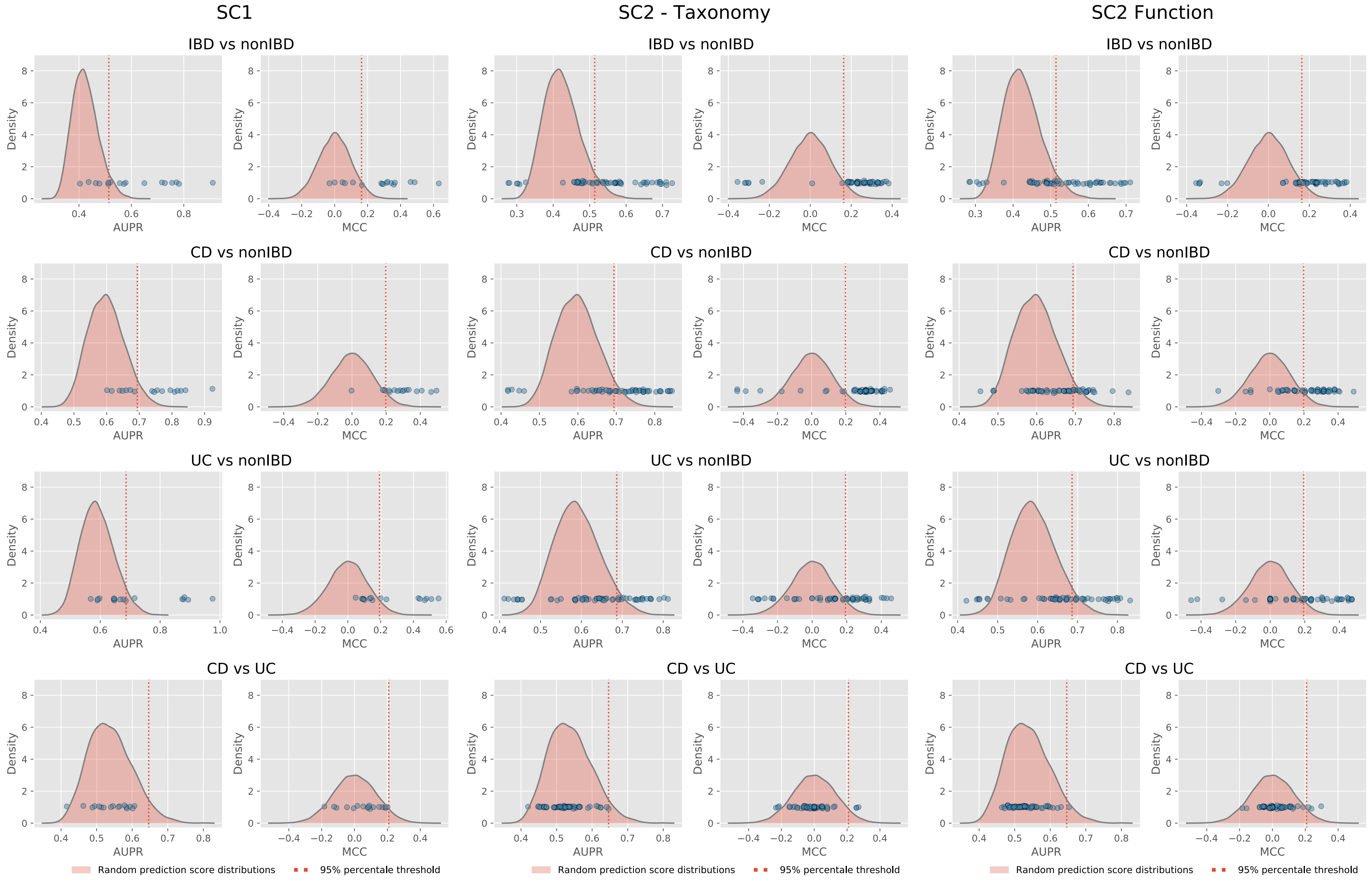

Supplementary Figure 2. Submissions ranking in the sbv IMPROVER MEDIC Challenge. Results are shown separately for each SC, data type (for SC2) and 2-class problem. MCC and AUPR metrics values are shown using the bar plots, with MCC/AUPR better than 95th percentile of the corresponding null distribution coloured in red and negative MCC better than 95th percentile of the corresponding null distribution colored in blue. Figure legend corresponds to the one on the Figure 2

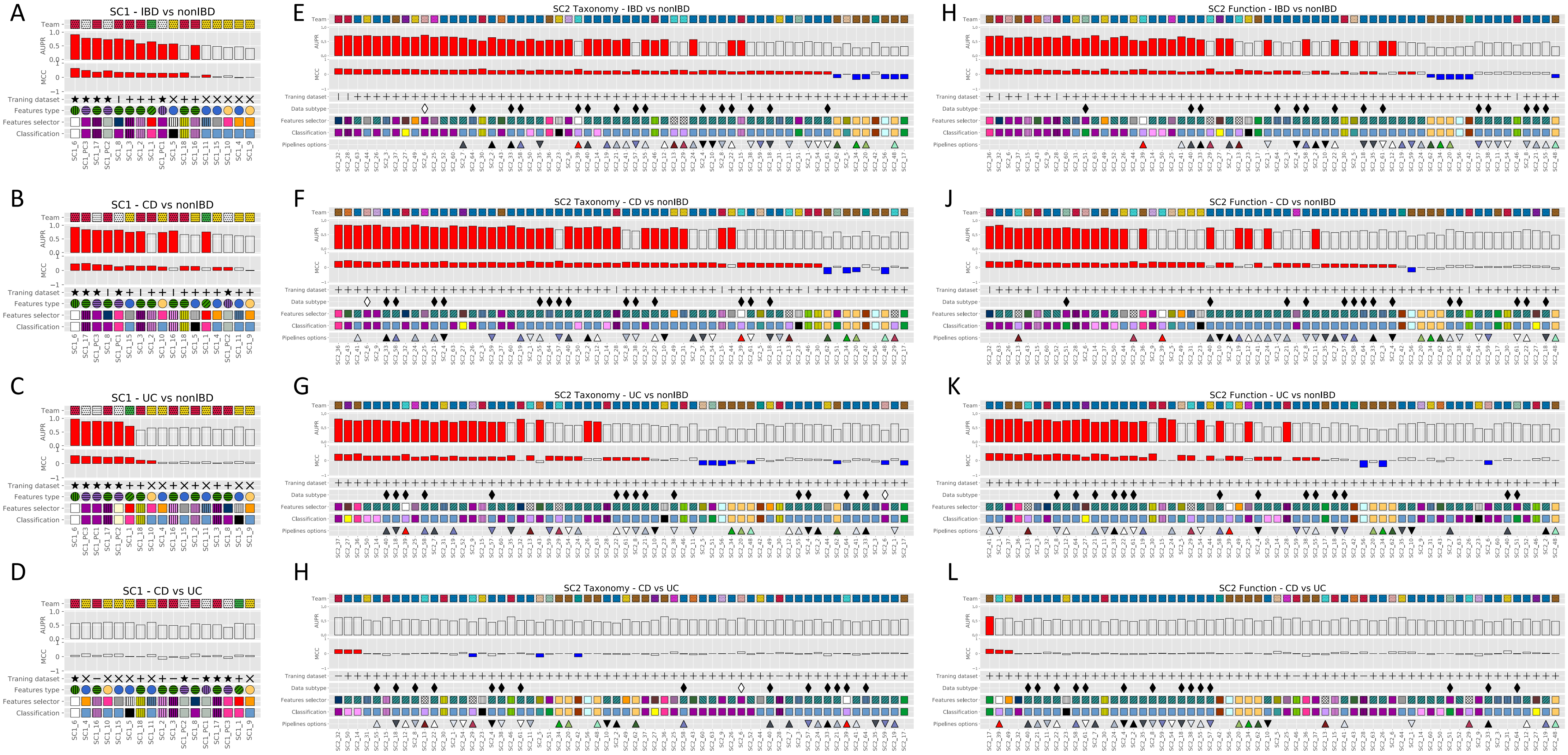

Supplementary Figure 3. Distributions of confidence scores retrieved from the participants submissions stratified by the sample label. Results are shown separately for each SC, data type (for SC2), 2-class problem, and metric. Submissions with MCC/AUPR better than 95th percentile of the corresponding null distribution are marked with black cross. Submissions with negative MCC higher than 95th percentile of the corresponding null distribution are marked with red cross.

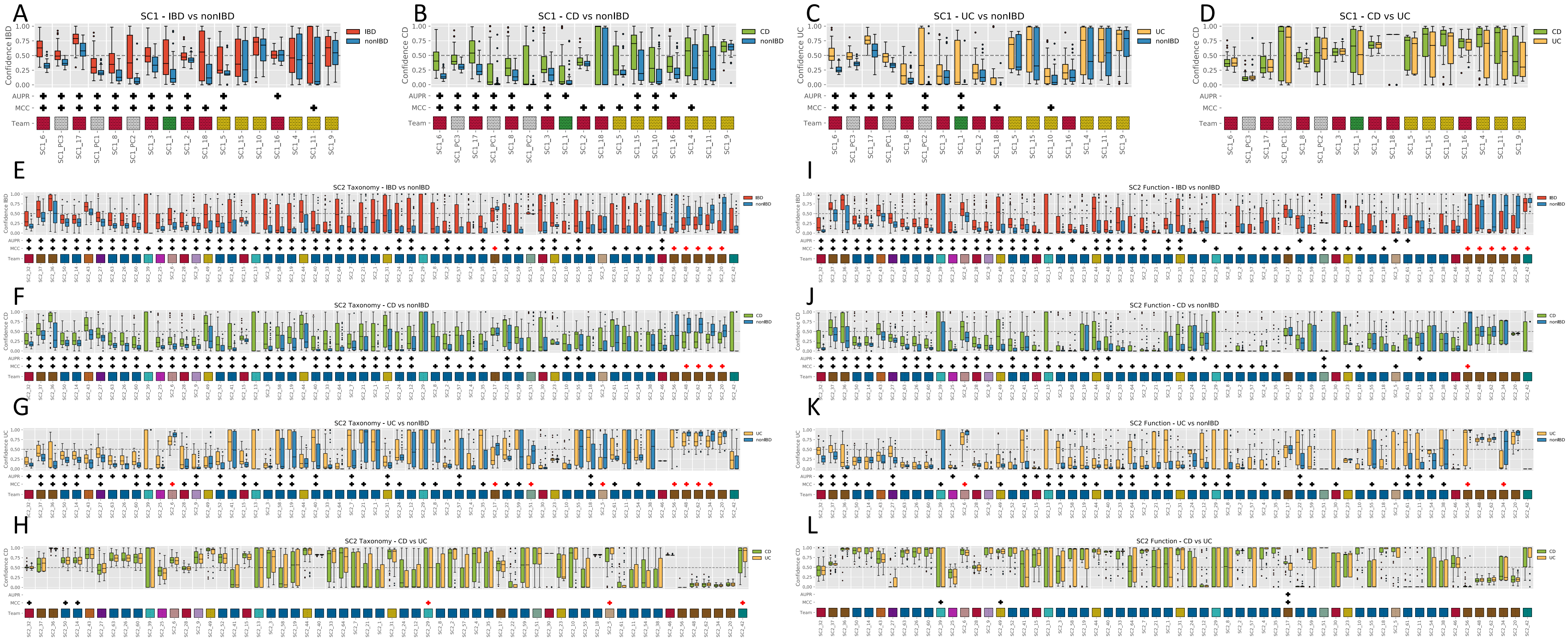

Supplementary Figure 4. Supplementary Figure 5 – clustering of absolute predictions, SC1.

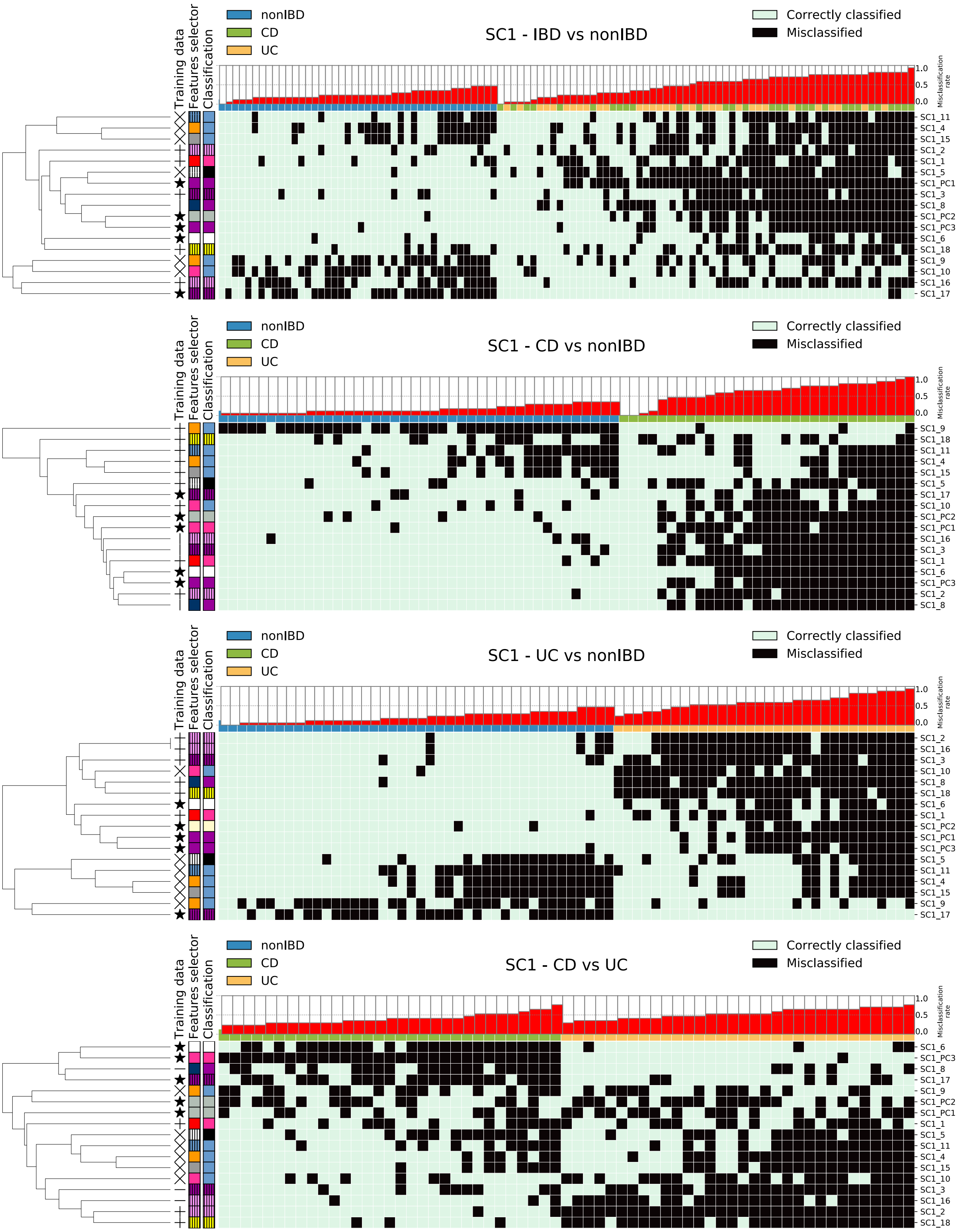

Supplementary Figure 5. Clustering of absolute predictions, SC2 Taxonomy, duplicated submissions are shown in red.

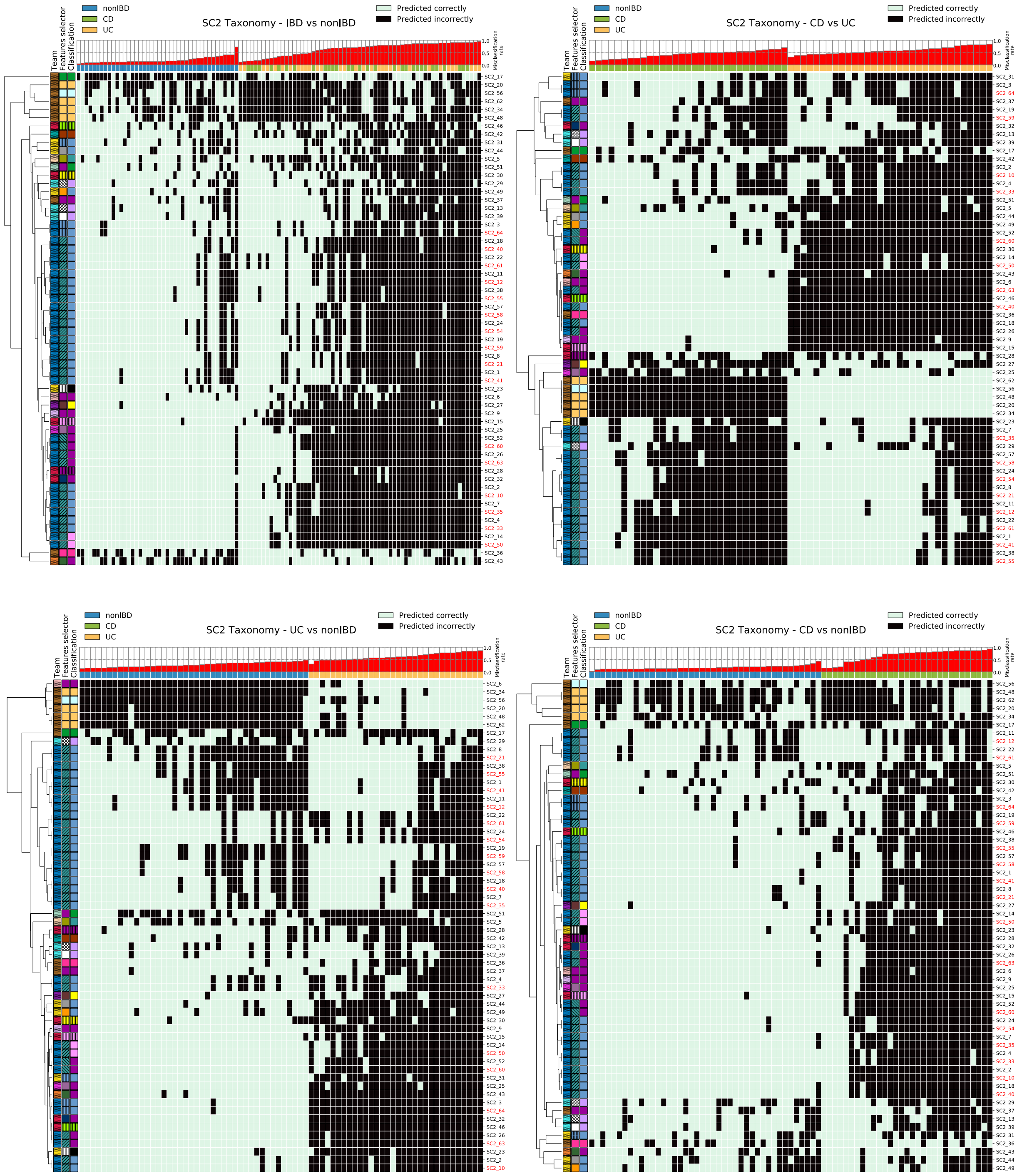

Supplementary Figure 6. Clustering of absolute predictions, SC2 Function, duplicated submissions are shown in red.

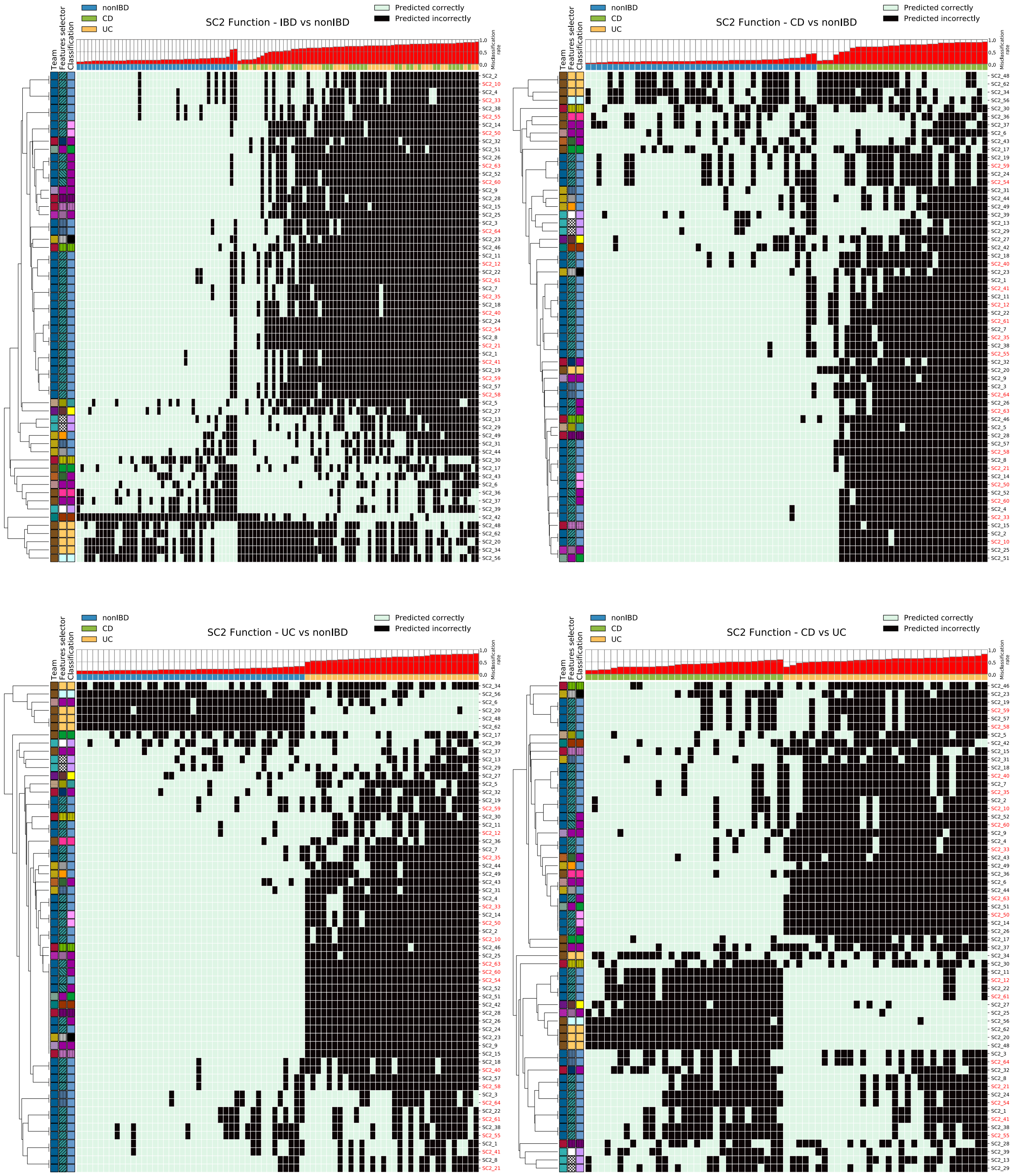

Supplementary Figure 7. Submission aggregation strategy.

Individual methods

	Method 1	Method 2	Method n	Combination
Sample 1	C_{11}	C_{21}	C_{n1}	$\frac{1}{n}\sum_{i=1}^{n} c_{i1}$
Sample 2	C_{12}	C_{22}	C_{n2}	$\frac{1}{n}\sum_{i=1}^{n} c_{i3}$
Sample 3	C_{13}	C_{23}	C_{n3}	$\frac{1}{n}\sum_{i=1}^{n} c_{i3}$
	…	…	…	…
Sample m	C_{1m}	C_{2m}	C_{nm}	$\frac{1}{n}\sum_{i=1}^{n} c_{im}$

Supplementary Figure 8. Analysis of assembly predictions vs. individual methods across different SC2 data types. Results for different 2-class problems and metrics are shown separately. First two boxplots represent the distributions of performances based on Taxonomy and Function data types, the third boxplot shows the distribution of performances obtained after assembling Taxonomy- and Function-based predictions across the same submission.

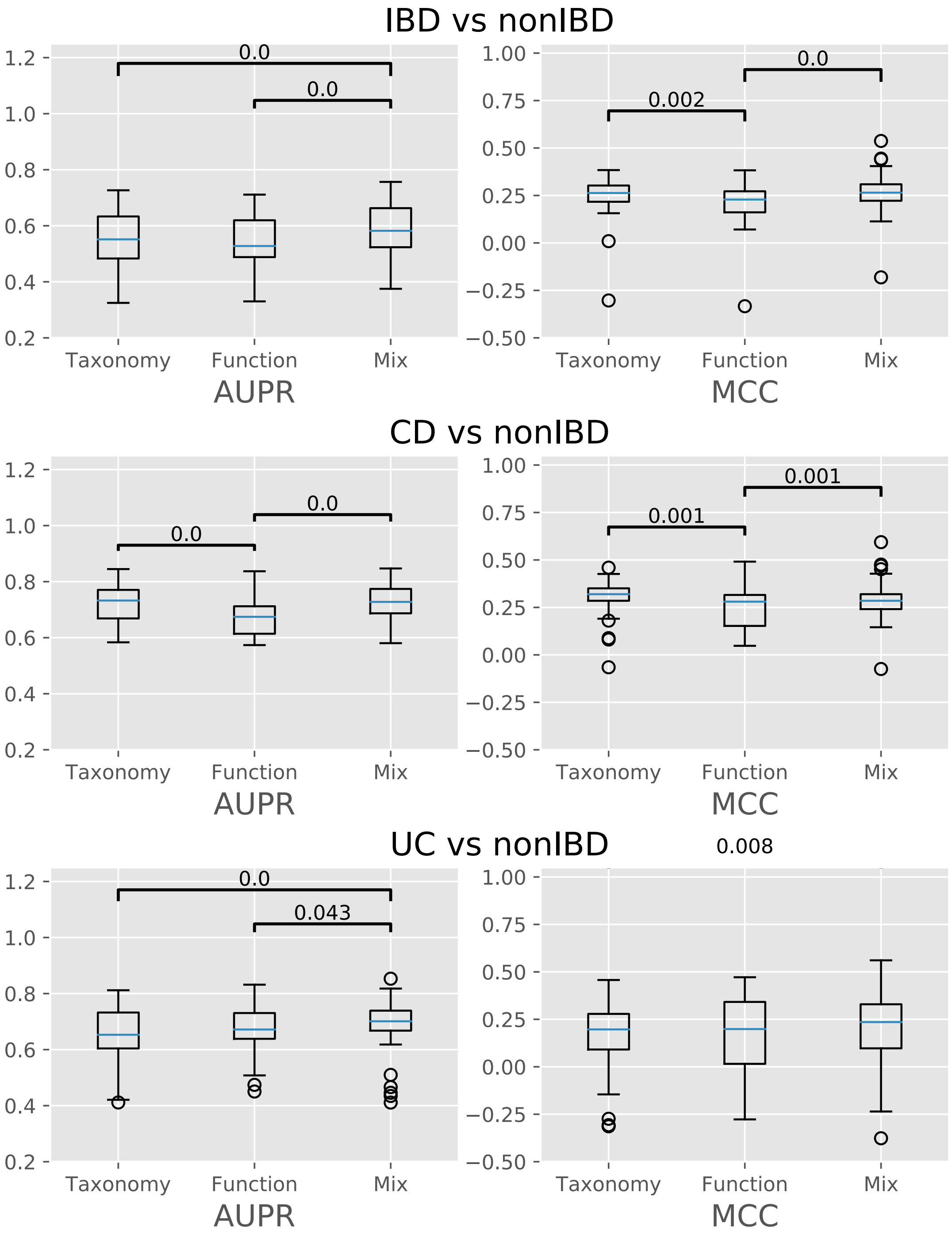

Supplementary Figure 9. Difference between mixed-based and individual methods. Results for different 2-class problems and metrics are shown separately. Each dot represents one submission, with its coordinates determined by the performance difference between mixed-based prediction and Taxonomy (Function) -based predictions. The dots color represents the performance difference between Taxonomy- and Function-based predictions

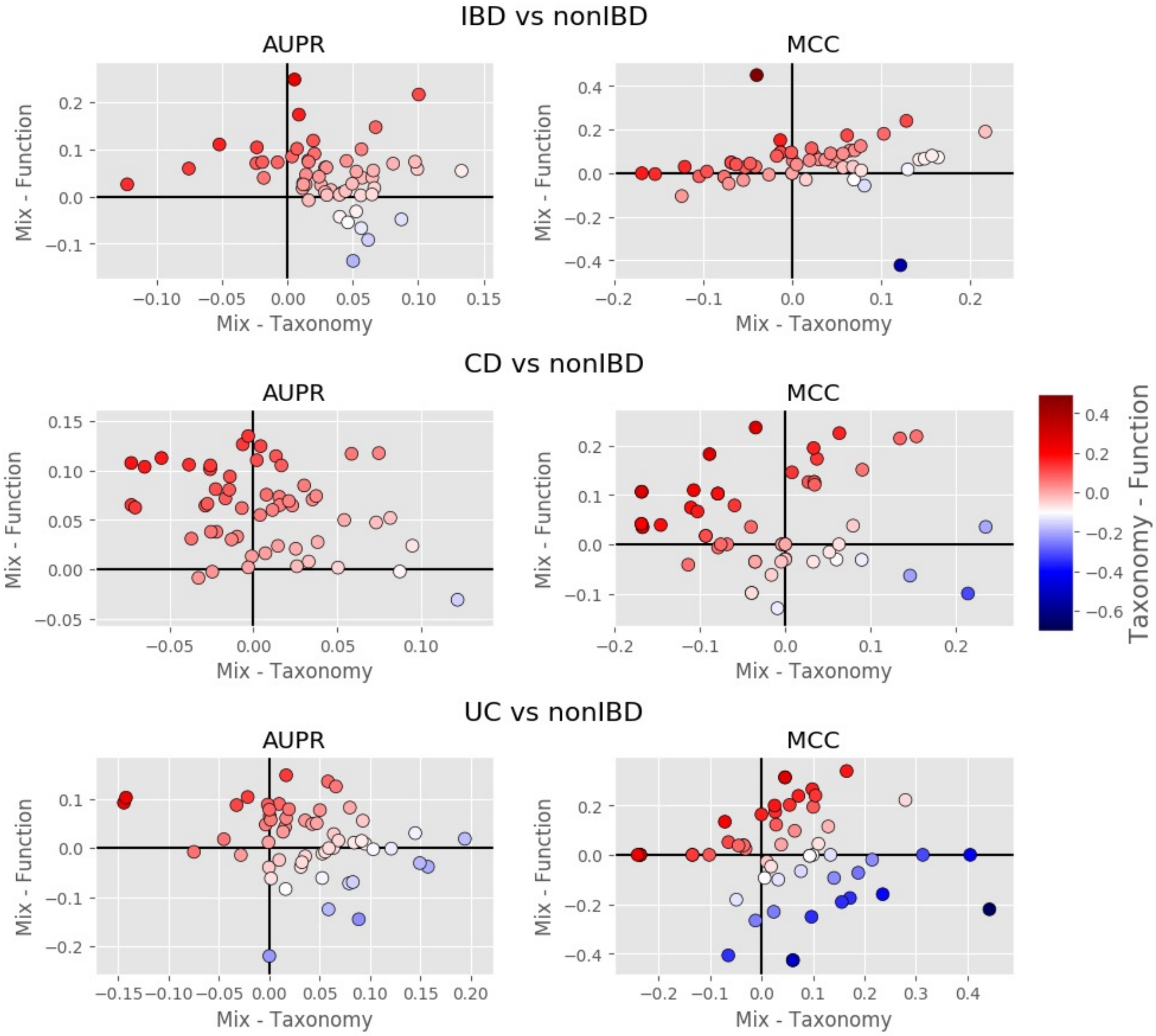

Supplementary Figure 10. Robustness of the assembly-based classification. Results for each SC, data type, 2-class problem and metric are represented separately. All individual predictions scores are shown with open circles (red for the best three, blue for the worst three and black for all others). The score for the prediction based on the assembly of all but the three best and three worst solutions ("initial" assembly) is shown with filled black dot. Results for the one-by-one adding of the worst three and best three methods to the "initial" assembly are shown with the line and filled dots in blue or red colour respectively.

SC1

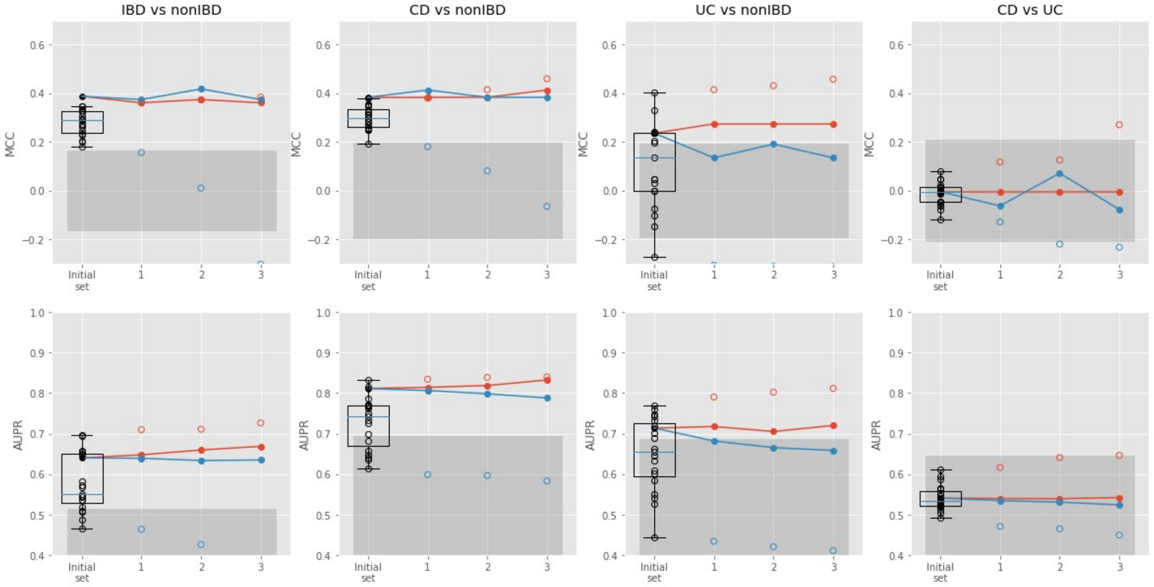

SC2 Taxonomy

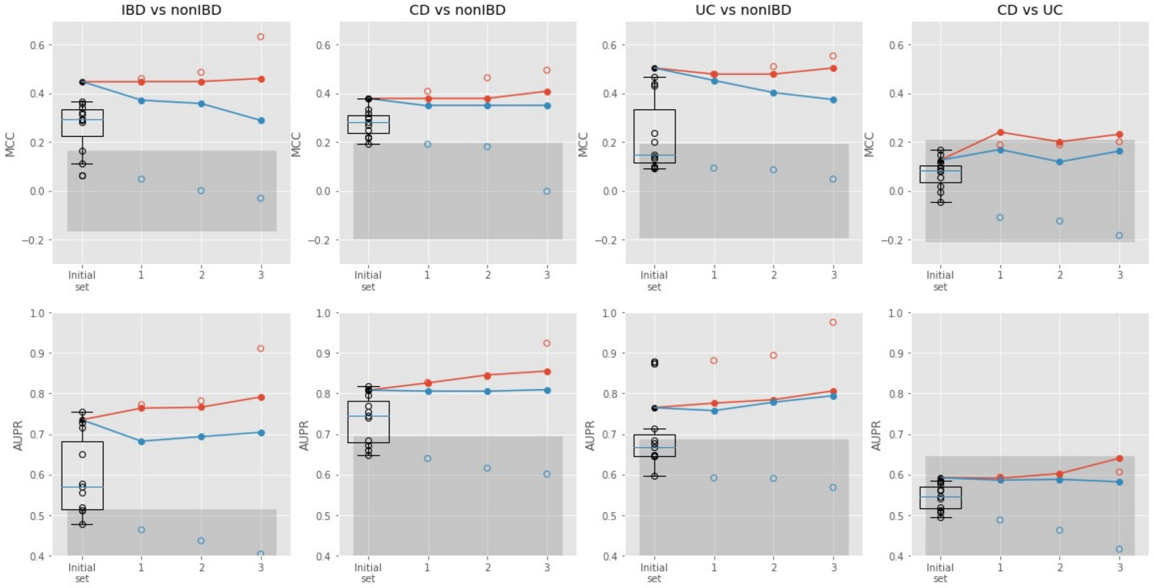

SC2 Function

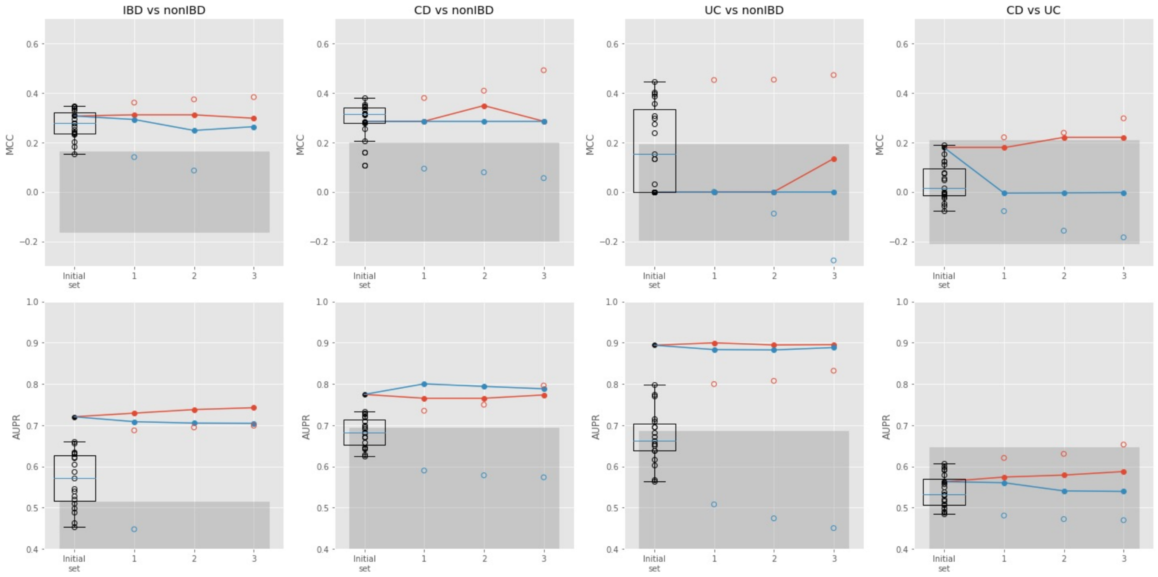

Supplement: Supplementary file 5 — Supplementary Information 5. [file 41598_2023_33050_MOESM5_ESM.pdf]
